# A biological blueprint for the axons of superficial layer pyramidal cells in cat primary visual cortex

**DOI:** 10.1007/s00429-017-1410-6

**Published:** 2017-04-07

**Authors:** Kevan A. C. Martin, Stephan Roth, Elisha S. Rusch

**Affiliations:** Institute of Neuroinformatics, UZH/ETH, Winterthurerstrasse 190, 8057 Zurich, Switzerland

**Keywords:** Visual cortex, Superficial layers, Pyramidal neuron, Axons, Bouton clusters, Local, Distal, Interbouton interval, Tortuosity

## Abstract

Pyramidal cells in the superficial layers of neocortex of higher mammals form a lateral network of axon clusters known as the ‘daisy’ network. The role of these axon clusters remains speculative and we still lack a comprehensive quantitative description of the single neurons forming the daisy or their heterogeneity. We filled intracellularly 50 superficial layer pyramidal neurons in the cat primary visual cortex and reconstructed the axonal tree and their synaptic boutons in 3D. Individual bouton clusters were identified using an objective mean-shift algorithm. By parameterizing the morphology of these 50 axonal trees and the 217 bouton clusters they formed, we were able to extract one set of relatively constant parameters and another set of variable parameters. Both sets combined allowed us to outline a comprehensive biological blueprint of superficial layer pyramidal neurons. Overall, our detailed analysis supports the hypothesis that pyramidal neurons use their lateral clusters to combine differential contextual cues, required for context-dependent processing of natural scenes.

## Introduction

In higher mammals, the pyramidal cells of the superficial cortical layers form a distinctive network consisting of a circular arrangement of clusters of synaptic boutons arranged around a core of cells, dendrites, and axon (Rockland and Lund 1982). Viewed en face, the network appears flower-like and has been referred to as the cortical ‘daisy’ (Douglas and Martin 2004). This daisy structure is made visible by extracellular injections of tracers. Analyses of the axons of individual superficial layer pyramidal neurons in visual cortex have shown how the daisy is built up from its components: a proximal or local bouton cluster forms around the dendritic tree. ‘Spokes’ then branch off in different lateral directions from the descending main axon to form the distal clusters (Gilbert and Wiesel 1983; Martin and Whitteridge 1984; Binzegger et al. 2007; Martin et al. 2014; Ojima et al. 1991). Each pyramidal cell axon contributes to only some of the distal clusters of the daisy, so that the full daisy is created by an ensemble of many pyramidal cells. The basic dimensions of this ensemble, measured in different areas and different species, reveal that a simple linear relationship exists between the size of the patches and the distance between them (Douglas and Martin 2004; Binzegger et al. 2004; Levitt et al. 1993, 1994; Lund et al. 1993; Muir et al. 2011). Binzegger et al. (2007) demonstrated that such a relation is not just confined to the superficial layer pyramidal cells, but applies to all cell types examined in cat V1, including smooth neurons. These observations give encouragement to the notion that the cortical daisy emerges from elemental rules that govern the growth of all cortical neurons.

**Fig. 1:**
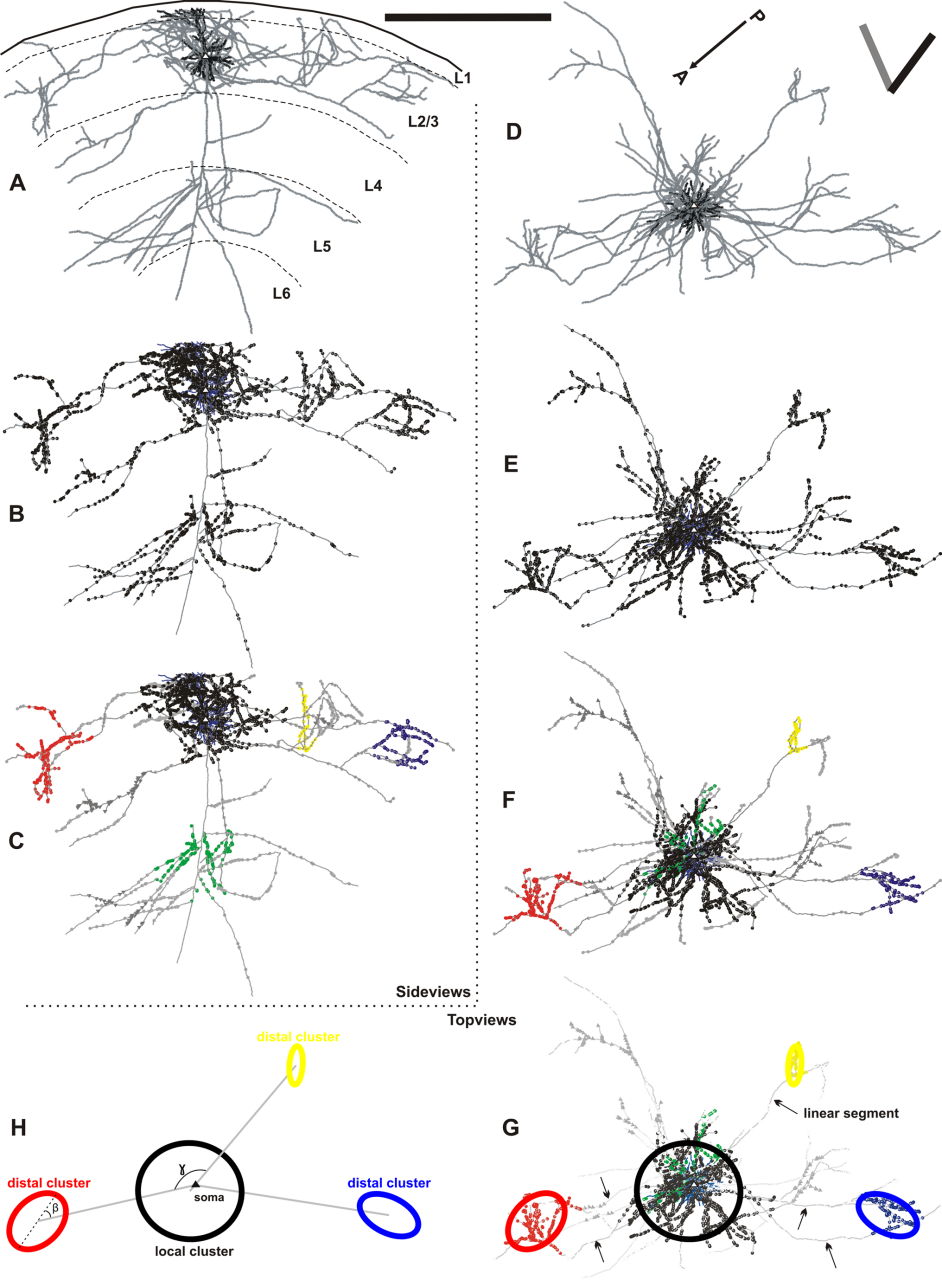
Example of a typical superficial layer pyramidal neuron. **a**–**c** The side view and **d**–**h** the top view of the reconstructed axonal tree, boutons, dendrite and ellipsoids. **a** The brain surface and the layer boundaries are depicted with *black curves*. The axonal tree (*grey*) forms extensive lateral connections in the supragranular layers and minor bifurcations within layer 5. The dendritic tree (*black*) bifurcates locally and forms one apical dendrite towards layer 1 to form a tuft. **b** Axonal boutons are denoted by enlarged *black dots* forming high and low density regions within layers 2 and 3 and layer 5. These regions of high bouton densities are captured by the use of a mean-shift cluster-algorithm (applied on the 3D data, see Methods). **c** The cluster-algorithm extracted five discriminated regions of high bouton densities (termed as clusters). The boutons itself attributed to the five different clusters are indicated with different colors (*black, red, green, blue* and *yellow* in order of their corresponding increasing cluster size). Boutons outside clusters are marked in *grey*; boutons belonging to omitted clusters are highlighted by *grey triangles* (see “Methods”). **d** The top view of the axonal tree (*grey*) and the dendritic tree (*black*) are displayed together with the anterior–posterior axis, the vertical meridian (*black bar*) and the neuron’s preferred orientation (*grey bar*). **e** The boutons (*black*) are densely packed in the neurons vicinity and are clotted at distant regions roughly 1-mm away from the soma. These high-density regions, nicely captured by the cluster-algorithm (see **f**), are interleaved by sparse regions. **g** For simplification and further quantification ellipsoids were fitted to each individual bouton cluster and in the here shown 2D view represented by ellipses (see “Methods”). The *arrows* point to those radial axons extending *horizontally* to form clusters, termed here as *linear segments*. **h** Only the ellipses of **g** are shown to highlight the concept of the local cluster (*black*) and the 3 distal clusters (*red, blue, yellow*). Please see “Results ” and “Methods” for angle *beta* and *gamma*. (Neuron ID: 17, cat_1007_RH_neuron_01). *Scale bar* 1 mm

At single cell resolution, the arrangement of one single local and multiple distal clusters is a characteristic of superficial layer pyramidal in all areas of higher mammalian neocortex so far examined (Gilbert and Wiesel 1979; Juliano et al. 1989; Matsubara and Phillips 1988; Wallace and Bajwa 1991; Ojima et al. 1991; Yabuta and Callaway 1998; Kisvarday et al. 1986; McGuire et al. 1991; Martin and Whitteridge 1984; Binzegger et al. 2007). The local cluster around the dendritic tree is typically the largest in extent and contains the most boutons (Fig. 1h reiterates the concept of local versus distal). The distal clusters vary in number from cell to cell and for an individual cell, no two clusters are the same size or contain the same number of boutons (Kisvarday and Eysel 1992; Binzegger et al. 2004, 2005, 2007). When ranked according to the number of boutons in each cluster, the distribution of boutons across the clusters is seen to follow an exponential distribution (Binzegger et al. 2007). These morphological observations indicate that the placement of the distal clusters is not arbitrary. One obvious question follows: what determines the placement of the distal clusters?

In the visual cortex, the positioning of the distal clusters is commonly thought to be the means whereby domains of similar orientation preference are linked (Gilbert and Wiesel 1989; Bosking et al. 1997; Malach et al. 1993, 1994; Sincich and Blasdel 2001). Thus one interpretation is that the daisy pattern is driven by the functional need to achieve a ‘like-to-like’ rule of connectivity, perhaps by a Hebbian ‘fire-together–wire-together’ mechanism. At single cell resolution; however, we found strong evidence of a more complex pattern of connectivity (Martin et al. 2014). Superficial pyramidal cells did not form their distal clusters exclusively in domains that had the same orientation preference as those of the parent dendritic tree and local cluster. Instead, distal clusters were found in a variety of different domains, including orientation domains that had orientation preferences orthogonal to that of the parent dendritic tree (Martin et al. 2014). This architecture seems well-suited for context-dependent processing. Indeed, such context dependence of the daisy network is evident at the level of receptive fields. By inactivating small regions of V1, the orientation tuning of neurons at distant sites (>400 microns), can be shifted (Girardin and Martin 2009) or broadened, and direction selectivity can be lost (Crook et al. 1997, 1998). Similarly, there are many illusions in which a straight line appears to be bent due to the context of the lines—the Hering Illusion is one prominent example of these effects, which arguably may be a natural expression of the daisy architecture.

Despite such a long history of studies of the cortical daisy, we still lack a comprehensive and general description of the mesoscopic (i.e., light microscopic resolution) structure of the axons of the individual neurons that actually form the daisy. We thus embarked on a fine-grained analyses of a unique set of 50 superficial layer pyramidal cells from cat V1 that we had obtained by intracellular labeling during electrophysiological and optical imaging studies. Despite a high degree of individual variation in the axonal arbors, it was clear that these neurons all belonged to the same ‘family’ and thus we were able to identify a number of attributes that describe characteristic features of the organization of their axonal arborisations.

## Results

To define the ‘bauplan’ of the cortical daisy by describing a biological blueprint of superficial layer pyramidal neurons, we collected and analyzed a substantial number of neurons. Out of 231 recording sites, we attempted to label intracellularly 153 individual neurons. Their average receptive field size was 1.7° × 1.9° at an average eccentricity estimated at 3.1° from the area centralis. Of the 153 impaled neurons, we recovered 45 pyramidal cells from layers 2 and 3 and 5 star pyramidal cells from layer 4 that had well-filled dendritic and axonal arbors. Figure 1 shows one typical example of the dataset. Here the cell has been reconstructed from serial sections made in the plane of the optical imaging, which was near-horizontal in the stereotaxic plane and here called the ‘top view’. The conventional view of neurons is in the transverse view of the dendrites (black) and axon (grey) shown in Fig. 1a, which also allows the laminar boundaries to be indicated. In Fig. 1b, e the boutons along the collaterals of the axon are accentuated in size.

Historically, clusters in the axonal arborisations were defined subjectively by eye from from 2D projections or even single tangential sections. This subjective classification is unsatisfactory for quantitative analyses in 3D, particularly at single cell resolution, so we have developed a mean-shift algorithm to provide a more objective method of identifying clusters in 3D (Methods and Binzegger et al. 2007). The local cluster is indicated in black and the distal clusters in rainbow colors in Fig. 1c, f–h. The cluster in the deep layers (boutons colored green) lies radially beneath the parent cell body.

A cluster was assigned to a ‘home’ layer if more than 50% of its boutons were in that layer. Twenty-eight out of 217 clusters across all neurons were in layer 1, 157 in layers 2 and 3, 4 in layer 4, 24 in layer 5, and 4 in layer 6. Ellipsoids were fitted to the clusters identified by the mean-shift algorithm (“Methods”; Fig. 1h, g; long axis dotted line in Fig. 1h). They provide a means of determining the centre of the clusters, which then allowed us to measure the angles at which the distal clusters were located relative to the cell soma, as shown in Fig. 1g, h for the top view. The size of the ellipsoids also emphasises the differences in the sizes of the clusters. The distal clusters are linked to the parent cell by linear segments of axon (arrowed in Fig. 1g). Of the 50 neurons examined, the home layer of the local cluster was twice layer 1, 47 times layers 2 and 3, once layer 4, and never layers 5 or 6. The pyramidal neurons innervated their home layer with 64% of all their boutons and 72% of all their clusters. The remaining clusters were located in the deep layers.

**Fig. 2:**
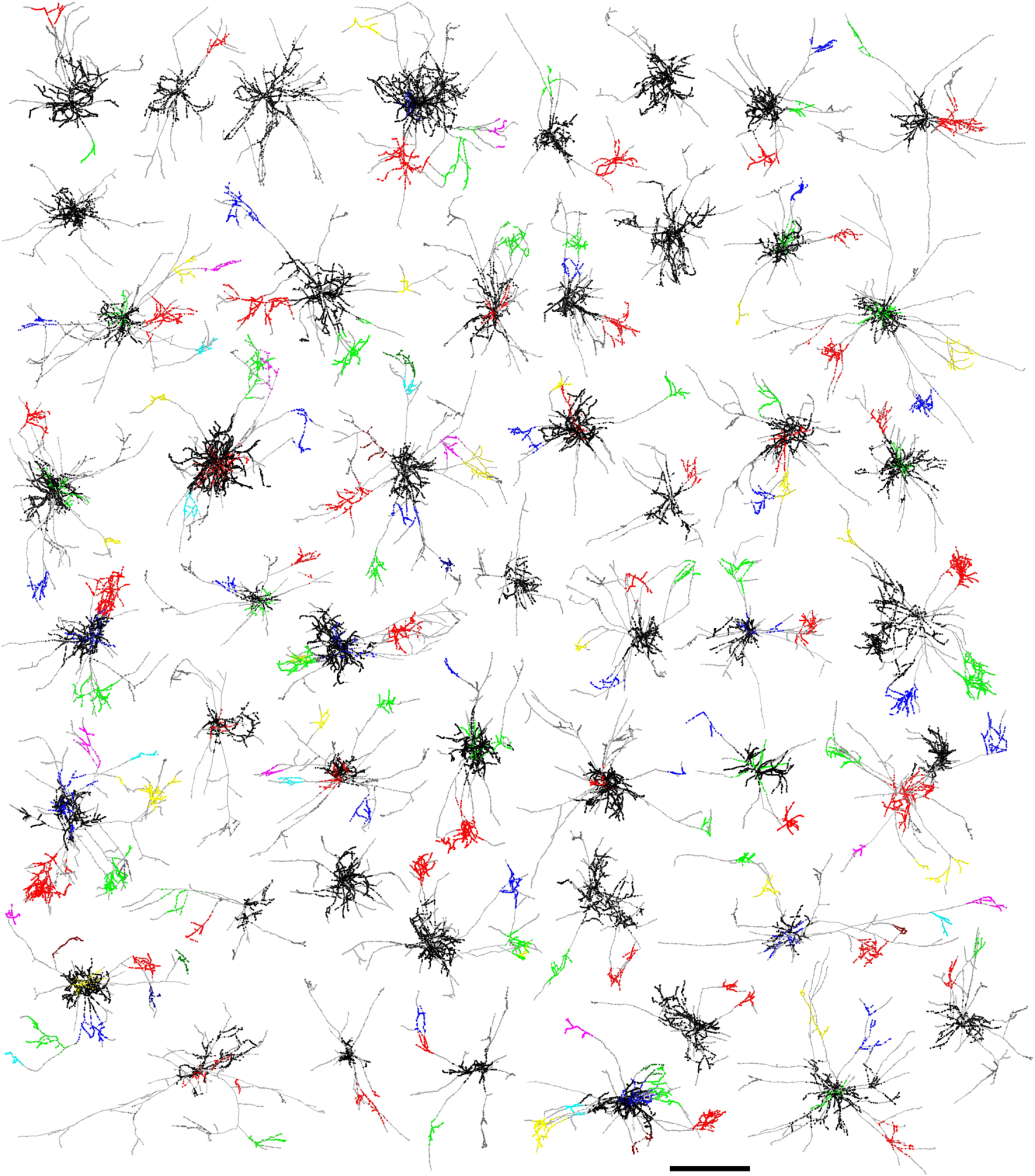
All 50 superficial layer pyramidal neurons of the dataset. *Top view* of the 50 superficial layer pyramidal neurons used in this study. Axons and non-clustered boutons are shown in *grey*. Clustered boutons are colored according to their rank (see Fig. 1 for color conventions). Neurons are sorted from *top left* to *bottom right* by normalized depth of soma (see Fig. 3 *top*). Each neuron was individually rotated that the empty space between was used best. Note that the neurons are shown in their entirety (incl. deep layer processes) whereas the actual study investigates only those parts in the superficial layers. *Scale bar* 1 mm

Figure 2 shows the top view of the axons of all 50 neurons used in the analyses. The top view indicates clearly the family resemblances between the different cells. These resemblances include a relatively large local cluster, with a small number of spoke-like linear axons that radiate from the center to end in smaller distal clusters. Note that it is rare for any one of the radiating linear axons to form more than one distal cluster. Figure 2 also emphasizes the variances between individuals. Indeed, each axon is unique, which makes the definition of a ‘bauplan’ a formidable task, despite the family resemblances.

**Fig. 3:**
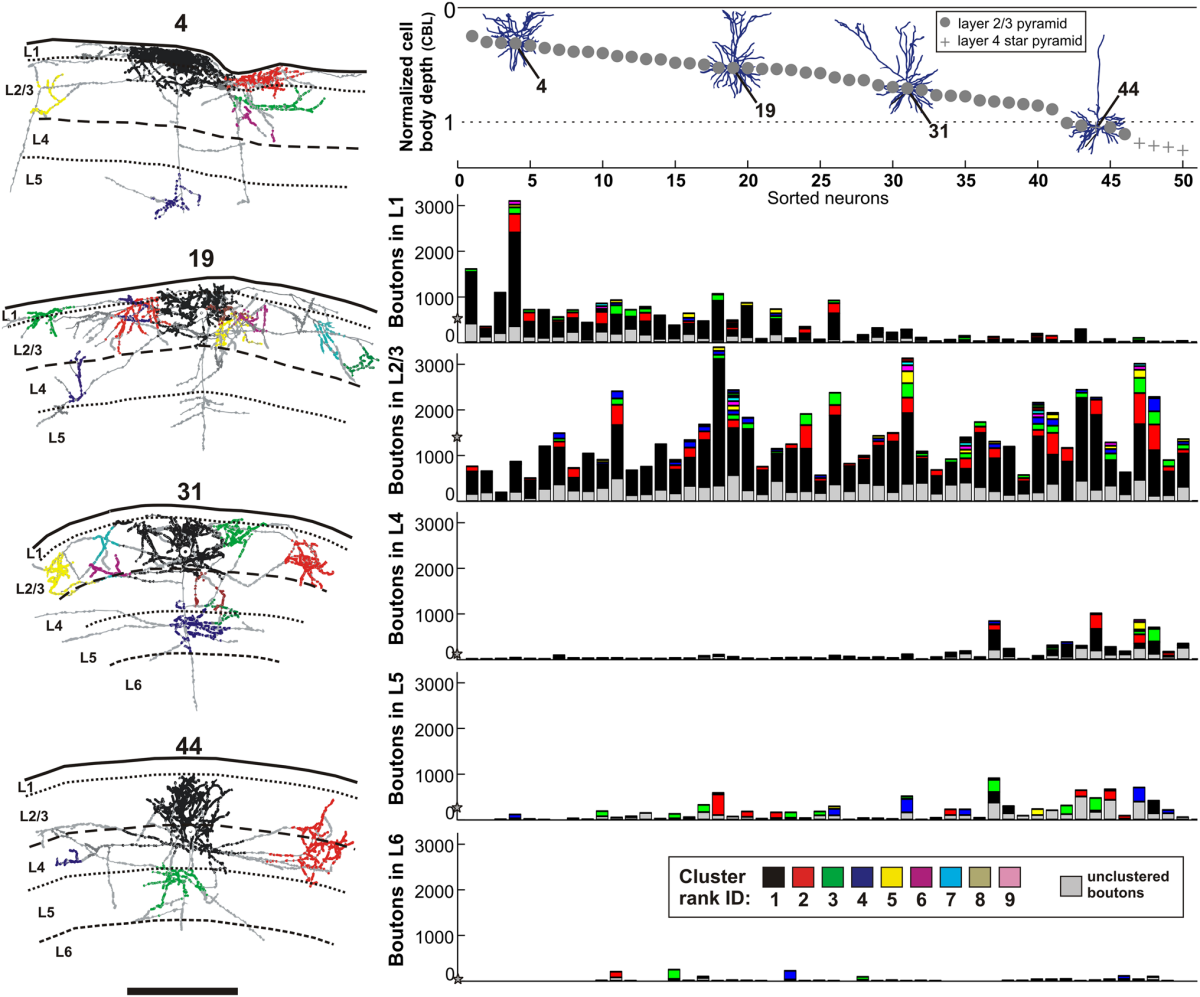
Laminar specificity of boutons. (*left*) Three examples of layer 2 and 3 pyramidal neurons (ID: 4, 19, 31) each located at a different depth from brain surface and one layer 4 star pyramid neuron (ID: 44). The brain surface and the layer boundaries are depicted with *black curves*. Boutons are color-coded by their cluster membership (*black, red, green, blue, yellow*, etc. in order of their corresponding increasing cluster size) and are given ascending cluster ranks (1, 2, 3, 4, etc.). Boutons outside clusters are marked in grey. The first neuron (ID 4) is located close to the border of layer 1/2 and the majority of its boutons are within layer 1. The somas of the second and third neuron (ID 19, 31) are in the *upper* and *lower half* of layers 2 and 3 and the majority of their boutons are within layers 2 and 3. Additionally, neuron 31 forms less boutons in layer 1 than neuron 19 and comparable more boutons in layer 5. The forth neuron (ID 44), a layer 4 star pyramid, forms a substantial fraction of boutons within layers 2 and 3 and 4. The dendrites of all four neurons are shown in the panel up right (*up right*) Normalized depth from surface (*y*-axis) for each of the 50 neurons: 45 superficial pyramids (*grey dots*) and 5 layer 4 star pyramids (*grey pluses*). The neurons are sorted by their increasing depth from surface giving each neuron a unique ID between 1 and 50 (*x-axis*). The brain surface is marked with a *solid black line* (depth = 0) and the border of layer 3/4 by a dotted line (border layer 3/4 = 1). (*Right*) Five histograms are shown for each of the 5 lamina (L1, L2/3, L4, L5 and L6). The *x*-axis reflects the IDs of the sorted neurons and the *y-*axis indicates the number of boutons encountered within each layer. The layers and their affiliated boutons were volumetrically determined (see “Methods”). The individual bars for each neuron and each layer are further subdivided by the number of boutons a certain cluster forms within each layer. The *color code* for each cluster is shown at the *bottom*. The mean number of boutons per layer is depicted with a *black star* placed on the *y-*axis (Neuron ID: 4, cat_1007_RH_neuron_02; 19, cat_0408_RH_ neuron_01; 31, cat_0608_RH_neuron_06; 44, cat_0108_RH_neuron_01). *Scale bar* 1 mm

### Laminar specificity of boutons

The clusters identified by the mean-shift algorithm were ranked according to the number of boutons they contained. The local cluster typically contained the largest number of boutons and was assigned the rank 1. In only two cases was the rank 1 cluster not the local cluster. When ordered according to depth (Fig. 3, top right), the cell bodies of the 50 neurons were seen to be distributed evenly through layers 2–4. The transverse view (Fig. 3, left hand column) shows cells at different depths suggested that the boutons clusters they formed did not fill the entire depth of the layer, but were also stacked at depths in relation to the depth of the cell body, i.e., the ‘cell body location’ (CBL). This relation was investigated quantitatively by counting the boutons that the individual neurons formed in each of the layers 1–6. The results for each individual neuron are plotted in Fig. 3 (right histograms, color code of cluster rank indicated) and this analysis indicates that bouton distributions were also layered in relation to their parent cell body. Apart from layer 6, a significant correlation existed between the CBL and the number of boutons in that specific layer (layer 1, *r* = −0.62, *p* ≪ 0.05; layer 2 and 3, *r* = 0.31, *p* = 0.029; layer 4, *r* = 0.67, *p* < < 0.05; layer 5, *r* = 0.51, *p* = 0.001). These data indicate that within the broad divisions of the six cortical layers, there are yet finer sub-laminar gradations of the bouton locations that are closely related to the depth in the layer of the cell body.

### The main axon

**Fig. 4:**
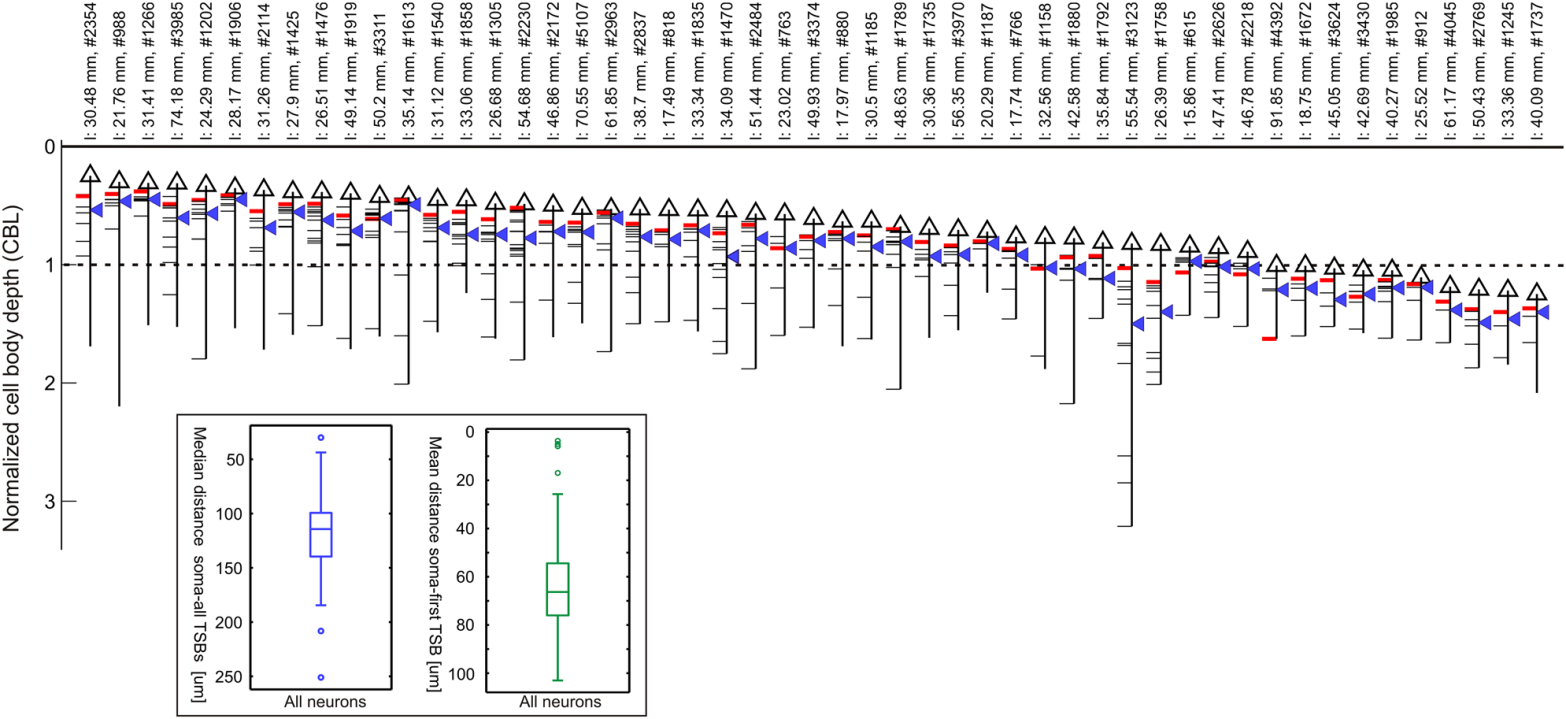
The trunk and the location of its branches. The neurons are sorted by their increasing depth from surface giving each neuron a unique ID between 1 and 50 (*x-axis*). The brain surface is at depth zero and the border of layer 3/4 is marked with a *dotted line* (border layer 3/4 = 1). Each neuron in the plot comprises a cell body (*black triangle*), a trunk (*black vertical line*) and several branches bifurcating from the trunk (*horizontal line* segments drawn to the *left*). These branches are termed as Trunk Side Branches (TSB). See also Fig. 5 for details. The trunk was cut below OT5 (see “Methods”) and the TSB contributing the highest fraction of boutons is marked in *red*. The median distance between the soma and the TSBs is denoted as a *blue triangle* to the *right* for each cell. Notice that on *top* of each cell the total length of its axon (l) and the number of boutons (#) are mentioned. (*Inlet*) Two boxplots showing the median of totally all distances between a neuron’s soma to all its TSBs (*left*) and the mean of the distance between the soma to the first branch across all neurons (*right*). The upper and lower boundary of the *boxes* denote the 25th and 75th percentiles, the whiskers extend to the most extreme data points to be not outliers. Outliers are shown as *circles*. Both measurements are remarkably constant across all neurons, and do not depend on the cell body depth

The main axon emerged from the soma and passed radially through the grey matter, emitting side branches (termed here as ‘Trunk Side Branches’—TSBs) before entering the white matter. Each TSB was numbered in order from the first TSB nearest the cell body. Figure 4 gives the neuron identifier and total axon length above each schematic neuron soma (triangles). The inset shows the median distance from the soma to all TSBs (indicated as blue triangle to the right for each neuron), which was 142.4 ± 46.5 µm (range 43.4–296.8), and the mean distance from the soma to the first TSB (indicated as the first tick to the left), which was 74.3 ± 25.3 µm (range 5.5–114.6). Both distances were independent of the position of the soma within the layers. The first TSB was formed on average after 74 µm from soma regardless of the depth of the soma below layer 1. By 142 µm half of all TSBs were formed (see Fig. 4 inset). The number of TSBs for each neuron was 7.8 ± 3.0 (range 3–16) within layers 1–4. There was a negative correlation (*r* = −0.55, *p* ≪ 0.05) between the number of TSBs and the depth of the cell body from the pial surface (i.e., CBL). This correlation showed that the deeper a neuron’s CBL, the fewer TSB’s were formed.

**Fig. 5:**
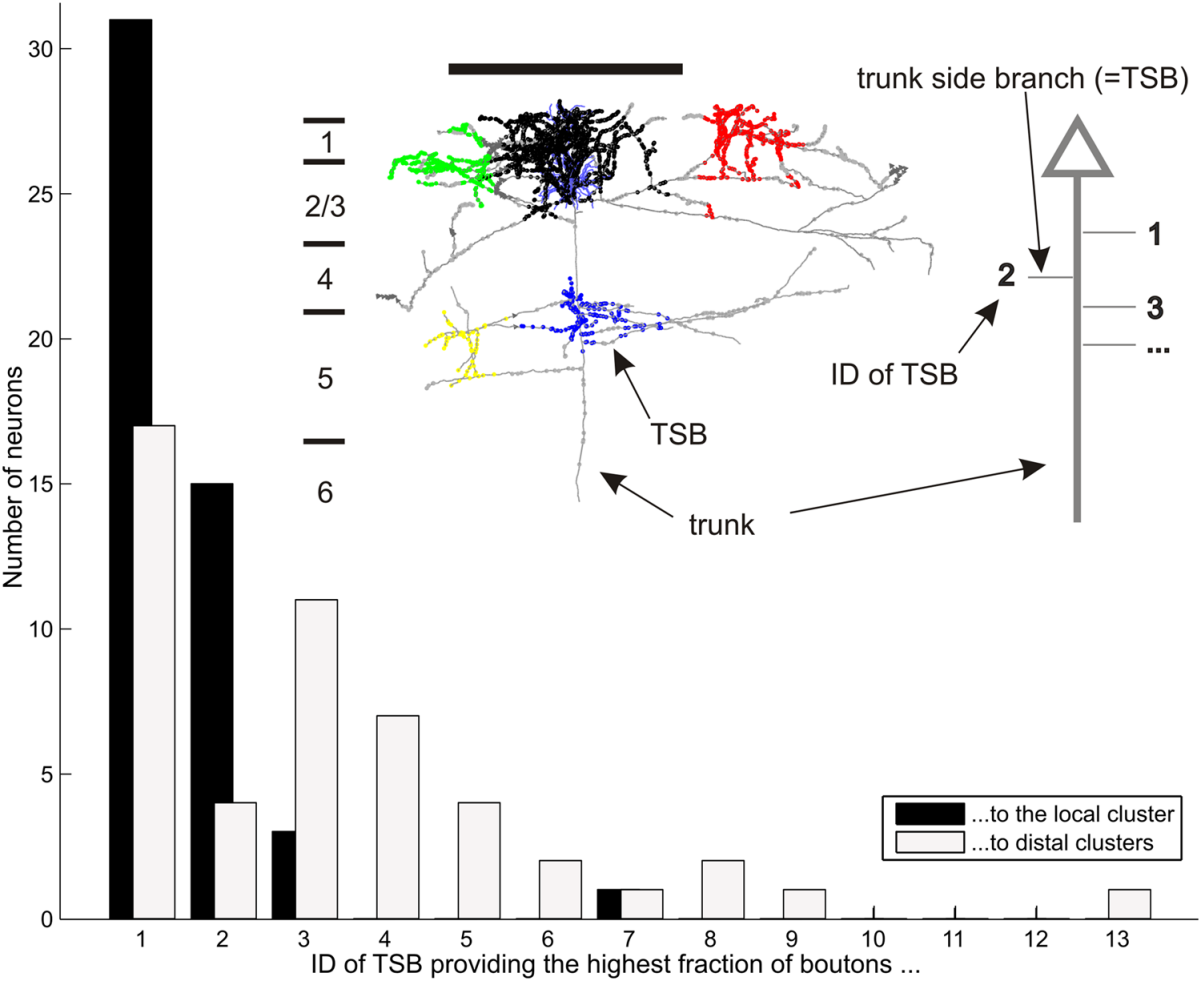
Distribution of trunk branch ID’s contributing the highest fraction of boutons to clusters. (*Inlet*) Example layer 2 and 3 pyramidal neuron with axon in *grey*, dendrite in *blue* and *color coded* bouton clusters (local cluster in *black*, 2 distal clusters in *green* and *red* and unclustered boutons in *grey*). Layer boundaries are shown on the *left* of the neuron (L1-6). The *right* shows a schematic representation of the soma (*grey triangle*), the trunk (*grey vertical line*) and the trunk branches (*grey horizontal lines*). The trunk is termed as the part of axon which projects radially towards the white matter concomitant elongating along a microcolumn. Each single neuron, out of our 50 examples, comprised such a trunk branching in a stereotypic manner. The branches bifurcating from the trunk (trunk side branches = TSBs) are ascending enumerated (trunk branch ID). For each ID the total number of boutons that are contributed to the local and distal clusters were calculated. (*Bar graph*) Distribution of trunk branch IDs contributing the highest fraction of boutons to either the local (*black*) or distal clusters (*grey*). The first trunk branch provided in 31 neurons the highest number of boutons to the local cluster. In 15 neurons it was the second trunk branch. This was significantly different for the trunk branches providing boutons to the distal clusters, where in 11 neurons/7 neurons provided the third/forth trunk branch the highest fraction of boutons. (Neuron ID: 26, cat_0608_RH_neuron_01). *Scale bar* 1 mm

### The trunk branch and the location of the clusters

After observing this remarkable constancy of the position along the main axon of the TSBs, we analyzed how the individual TSBs contributed to the individual bouton clusters. The TSB that contributed the highest fraction of boutons to clusters are indicated with a red tick to the left for each of the 50 neurons in Fig. 4. For most neurons, it was evident that their first TSB contributed the highest fraction of boutons to the local cluster, whereas for the distal clusters, the first four TSB’s contributed the highest fraction of the boutons. This relationship is plotted quantitatively in Fig. 5, which shows that the first 3 TSBs contributed virtually all the boutons of the local cluster. Also apparent in Fig. 5 is the systematic decrease in the number of boutons contributed by successive TSBs.

### The contribution of branches originating in layer five

Superficial layer pyramidal neurons form most of their boutons in the region between layer 1 and the border of layers 3 and 4, with an additional arborisation in layer 5 (see histograms in Fig. 3). This division into two tiers of innervation is reflected in the TSBs along the trunk. Most TSBs were in layers 2 and 3, layer 4 was largely avoided and then a few TSBs were emitted in layer 5 (see example neuron in left panel of Fig. 5). As our main focus was on the components that form the daisy in the superficial layers, we then determined how many of the TSBs emitted in the deep layers contributed boutons to the clusters in the superficial layers. In turned out that this fraction was only 8.5 ± 11.9% (range 0.0–22.1%). Thus, all the TSBs below layer 4 could be excluded from further analyses with minimal impact on the outcome. The clusters in the deep layers (i.e., layers 5 and 6) were not analyzed further, except for the measurement of their interbouton intervals.

### The size of local versus distal clusters

**Fig. 6:**
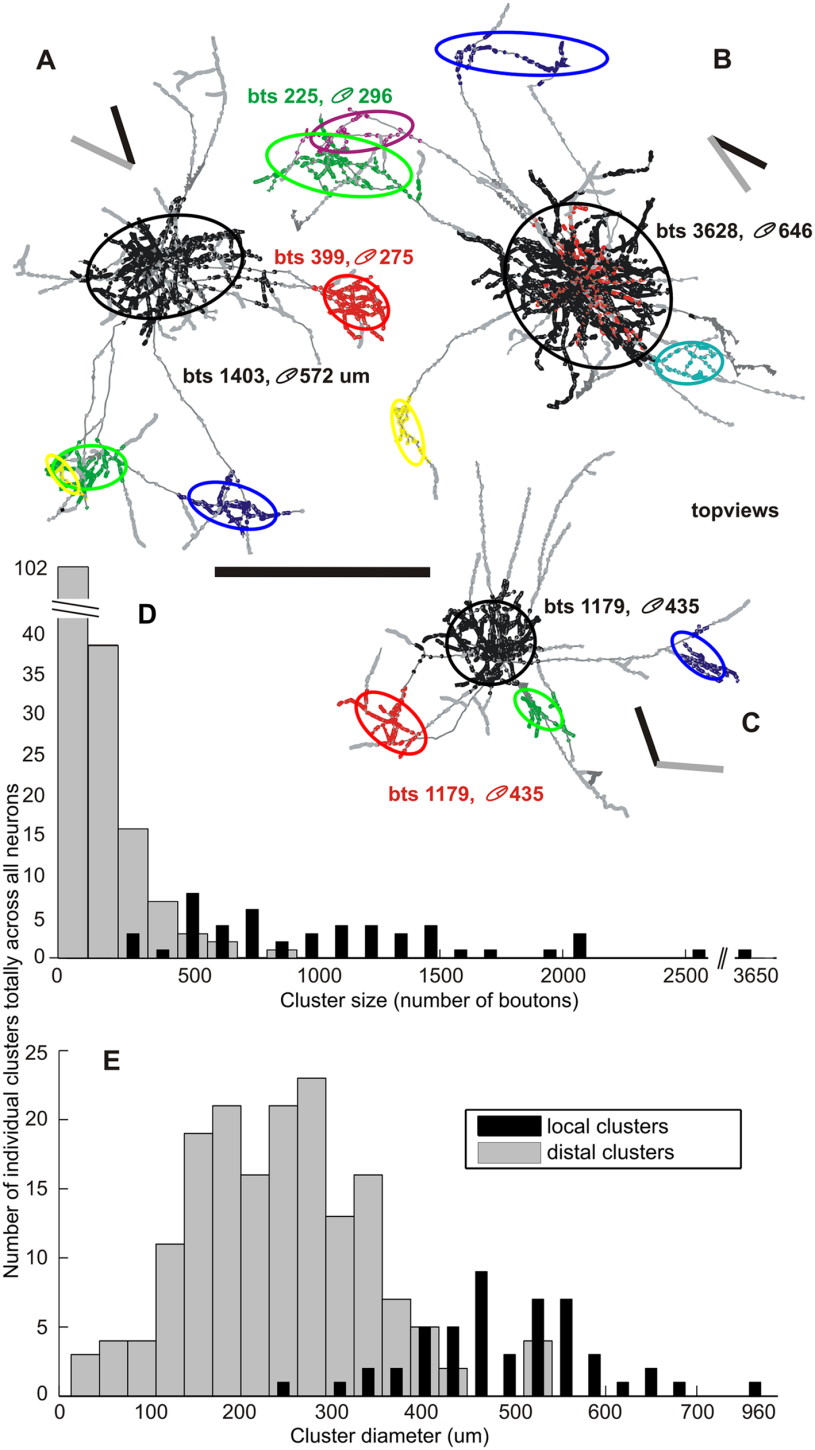
Distribution of cluster size and cluster diameter. **a**–**c** Three examples of layers 2 and 3 pyramidal neurons (ID: *A* = 37, *B* = 18, *C* = 7) each located at a different depth from brain surface. The neurons are shown in their *top views* together with their vertical meridian (*black bar*) and the neuron’s preferred orientation (*grey bar*). Boutons belonging to different clusters are color-coded by different colors (*black, red, green, blue, yellow*, etc., in order of their corresponding increasing cluster rank). Boutons outside clusters are shown in *grey*. For simplification and further quantification ellipsoids were fitted to each individual bouton cluster and here shown in 2D view represented by ellipses (see “Methods”). The number of boutons belonging to a certain cluster and the mean geometric diameter of the corresponding ellipsoid are noted for two clusters of each neuron. **d** Distribution of the cluster size versus the number of individual clusters across all neurons. The *grey bars* indicate the distribution across all distal clusters (mean 166 ± 139, range 53–884) and the *black bars* across all local clusters (mean 1079 ± 547, range 270–3630). Notice that the local clusters are those with the rank 1 (*black*, as seen in **a**–**c**). **e** Distribution of the mean geometric cluster diameter versus the number of individual clusters across all neurons. The *grey bars* indicate the distribution across all distal clusters (mean 256 ± 97 µm, range 29–551) and the *black bars* across all local clusters (mean 511 ± 110 µm, range 274–960). Notice that doubling the cluster diameter means an eightfold bigger cluster volume. The local and distal clusters are eloquently different in terms of size and diameter and thus have to be investigated separately (Neuron ID: 7, cat_0907_RH_neuron_01; 18, cat_0608_RH_neuron_02; 37, cat_0707_RH_neuron_01). *Scale bar* 1 mm

Figure 6 provides a comparison of the size and bouton content of the local and distal clusters. Three exemplar neurons are illustrated in top view in Fig. 6a–c, with the projection of the ellipsoid (rank color coded as in key in Fig. 3). The number of boutons in a cluster varied across three orders of magnitude (Fig. 6d), but regardless of absolute number of boutons, the local cluster contained the largest fraction of boutons in all, but 2 neurons. The local cluster had an average diameter of 511 +/- 110 µm (range 274–960: Fig. 6e). The number of distal clusters averaged 3 (range 2–9 clusters per neuron, *n* = 217) and an average diameter of 256 ± 97 µm (range 29–551: Fig. 6e). Despite these size differences, local and distal clusters contained similar densities of boutons. The local clusters averaged 2.0 ± 0.9 boutons per 50 µm voxel [or 16,000 boutons/mm^3^] and the distal clusters 2.5 ± 1.9 [or 20,000 boutons/mm^3^].

### The weight of local versus distal clusters

Figure 6d highlighted the observation that individual clusters of each neuron had different numbers of boutons. We calculated the ‘weight’ of a cluster, which we defined as the fraction of the total of all boutons that a pyramidal cell produces (for more details see “Methods” and Binzegger et al. 2007). As expected from its relatively large size, the average weight of the local cluster was 0.70 ± 0.18 (0.30–1), compared to the mean for the distal clusters of 0.09 ± 0.06 (0.02–0.35). When ranked by weight, the number of boutons in the clusters for any given cell followed an exponential distribution, as previously described by Binzegger et al. for a range of different cell types in cat V1 (Binzegger et al. 2007).

### The interbouton interval

**Fig. 7:**
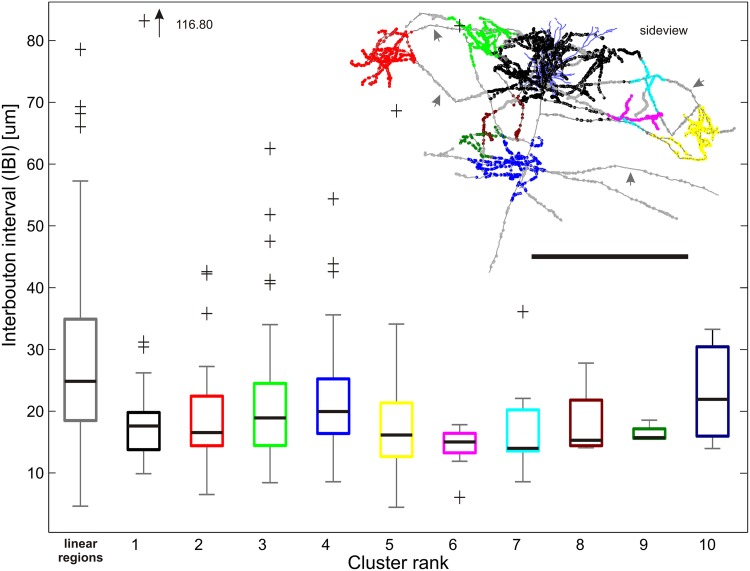
Interbouton interval (IBI) for clustered and linear regions totally across all 50 neurons. On the *x-axis* are the sorted cluster ranks listed (1–10, in their corresponding color) together with the linear regions (*grey*). The *y-axis* measures the IBI for each region represented as boxplots. *Boxplots* the *colored box* marks the lower and upper quartile, the *black bar* the median, the *whiskers* extend in both directions till the ×1.5 interquartile range and outliers are marked with *pluses*. The IBI is significantly higher in linear regions compared to each cluster rank (except for rank 10). The IBI does not significantly differ between different clusters (except for rank 10). (*Inlet*) Example neuron comprising 9 clusters colored according to their rank (compare to *x-axis*). The *arrows* point to examples on linear regions where the IBI is high, i.e. the axonal distance between two subsequent boutons is high (Neuron ID: 31, cat_0608_RH_neuron_06). *Scale bar* 1 mm

The similar bouton density in local and distal clusters suggested that the interbouton interval (IBI) might be equal across the whole axonal tree, as described by several investigations (Amir et al. 1993; Yabuta and Callaway 1998; Weller et al. 2000; Anderson et al. 2002; Karube and Kisvarday 2011). When sorted according to cluster rank, the IBI across all clusters is very similar, regardless of rank (Fig. 7). The IBI for local (17.3 ± 4.9 µm, range 9.5–30.6) and distal (19.7 ± 10.2 µm, range 5.7–68.5) clusters was not significantly different. The linear segments of axon, which link the clusters to the main axon, had a mean IBI that was significantly larger (28.3 ± 15.2 µm, range 4.3–82.4, Kruskal–Wallis, *p* ≪ 0.05), see Fig. 7 (left boxplot). Thus, the frequency of encountering a bouton along a linear axonal segment (3.5 boutons/100 µm) is significantly lower than in a cluster (5.4 boutons/100 µm).

### The clusters seen in top view

Seen from the top in 2D, the axons that form the distal clusters radiate like spokes from the soma and then branch into collaterals to form distal clusters (see examples in Figs. 1, 2, 6, 10, 11). Since a bouton cluster is well-fitted with an ellipsoid (see “Methods”), we explored the possibility that the elongation of the cluster was simply a reflection of the direction of outgrowth of the spoke. To test whether the clusters themselves elongate in a soma-eccentric manner (i.e., whether the semi-major axis of the ellipsoid fitted to the bouton cluster points towards the soma), we calculated an angle ‘*beta*’ between the ellipsoids semi-major axis and the axis of the Euclidean distance between the cluster center and the soma (see example Fig. 1h; 0 degree means soma-eccentric elongation). The angles *beta* had a mean of 50° ± 24° and ranged between 6.6° and 89°, indicating that individual distal clusters do not elongate along the soma–cluster axis (as seen for the three example neurons in Fig. 6a–c). Instead, they form a variety of angles, indicating that forces other than direction of outgrowth determine the long axis of individual clusters.

### Circular regularity in positioning of distal clusters

Since the ensemble of neurons can create a circularly symmetric daisy, we wondered whether any circular regularity exists when looking at the individual neurons in their top views. Circular regularity in this context can be interpreted as regularity between the minute and hour hand of a watch, e.g., displaying quarter hour interval. To describe circular regularities amongst the neuron’s distal clusters, we mapped them onto their projection plane (see “Methods”). For each neuron, lines were generated connecting all their distal cluster centers with their common soma. The clockwise angle ‘*gamma*’ between two lines of neighboring distal clusters (see example in Fig. 1h**)** provided the means of testing if there was any regularity. *Gamma* ranged from 2.6° to 335.8° with a mean of 106.4 ± 83.8. When pooling all neurons together, the distribution of *gamma* showed no regularity and no peak at any particular angle. This was still true when we pooled neurons with the same number of distal clusters or when we compared the angles between clusters with subsequent cluster ranks.

### Horizontal displacement of clusters

**Fig. 8:**
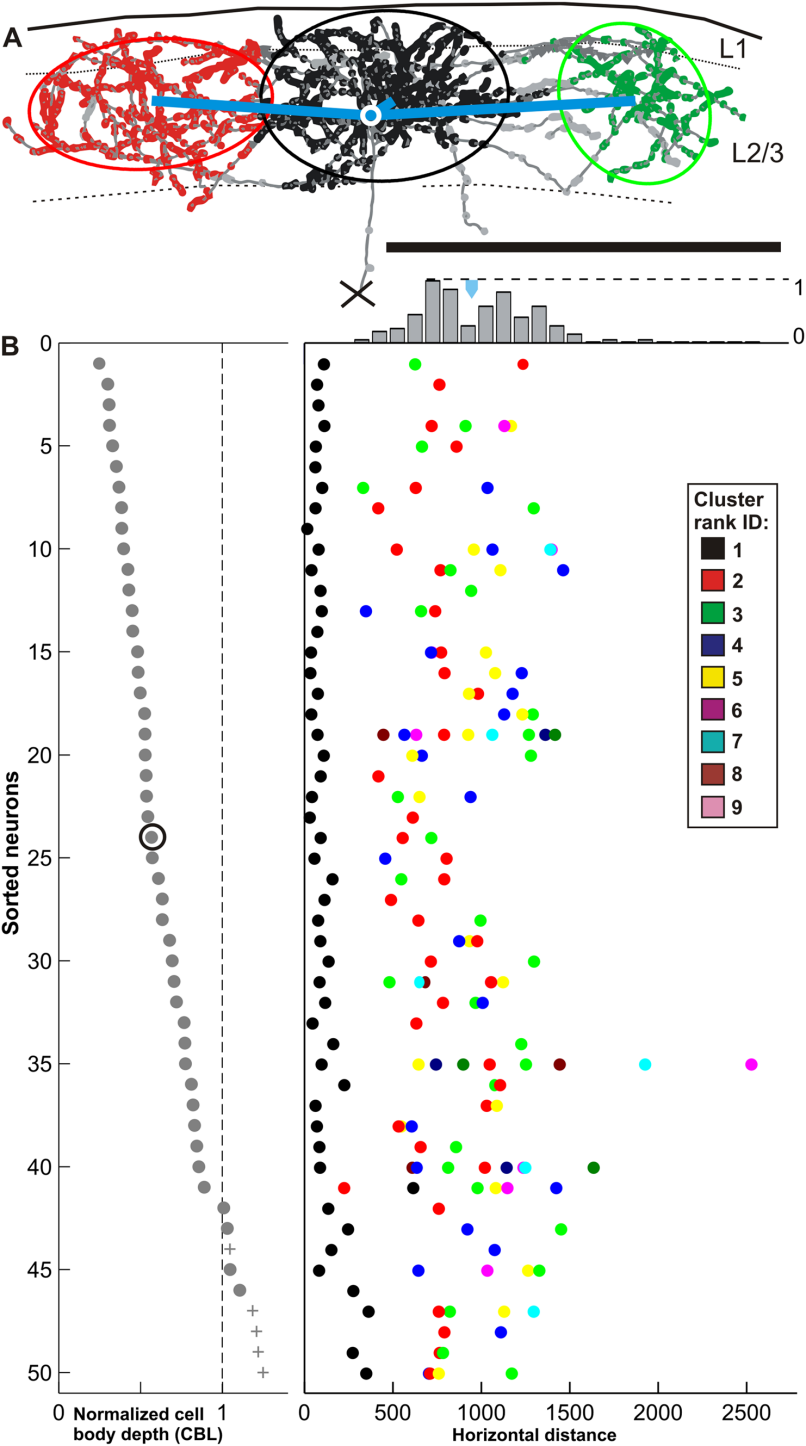
Horizontal distance of the superficial layer cluster center. **a** Example of a pyramidal neuron (ID: 24) located in the middle of layers 2 and 3. The deep part of the axonal tree was cut off at the marked location (*cross*). The brain surface and the superficial layer boundaries are depicted with *black curves*. The boutons’ cluster membership is color-coded with *black, red* and *green* in order of their corresponding increasing cluster rank. Boutons outside clusters are marked in *grey*. For simplification and further quantification ellipsoids were fitted to each individual bouton cluster, represented by an ellipse shown in 2D view (see “Methods”). The Euclidean distances between the soma (*white circle*) and each cluster center are highlighted by *blue lines*. (**b**, *left*) Normalized depth from surface (*x-axis*) for each of the 50 neurons: 45 superficial pyramids (*grey dots*) and 5 layer 4 star pyramids (*grey pluses*) are shown. The neurons are sorted by their increasing depth from surface giving each neuron a unique ID between 1 and 50 (*y-axis*). The brain surface is at depth zero and the border of layer 3/4 is marked with a *dotted line* (border layer 3/4 = 1). (**b**, *right*) Every Euclidean distance to each neuron’s cluster ellipsoid center (*x-axis*) is individually plotted for each of the 50 sorted neurons (*y-axis*). Each *colored* dot represents one cluster of a certain rank (see color-table) with its individual Euclidean distance. The example neuron 24 is marked with a *circle*. Notice that in 48 neurons the closest cluster is the biggest one (rank 1). The histogram (normalized to the maxima) indicates the total number of distal clusters having a certain Euclidean distance from soma (only distal clusters were considered). The *blue triangle* marks the mean across all distal clusters (942 µm) (Neuron ID: 24, cat_2806_RH_neuron_04). *Scale bar* 1 mm

The Euclidean distance between the soma and each distal cluster center (Fig. 8a), termed here as ‘horizontal distance’, was measured (942 ± 332 µm, range 231–2529 µm). Figure 8b plots the data for the local and ranked distal clusters for each neuron individually. The histogram (Fig. 8b top) gives the distribution across all 217 distal clusters and shows that the distal clusters effectively create an annulus around the local cluster.

### Radial displacement of clusters

**Fig. 9:**
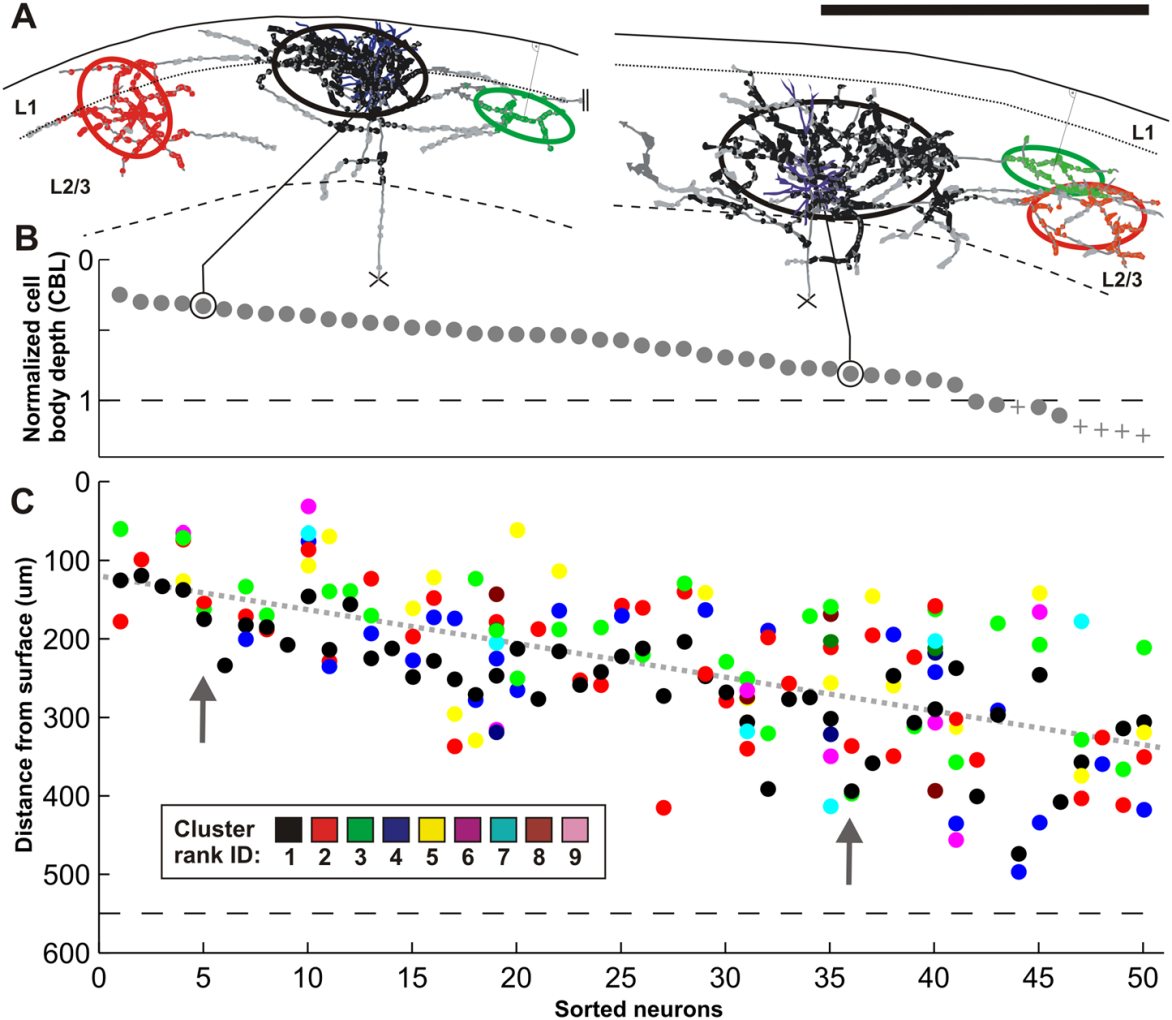
Vertical distance of the superficial layer cluster center. **a** Two examples of layers 2 and 3 pyramidal neurons (ID: 5, 36) each located at a different depth from brain surface. The deep part of the axonal tree was cut off at the marked location (*cross*). The brain surface and the superficial layer boundaries are depicted with *black curves*. Boutons belonging to different clusters are *color-coded* by different colors (*black, red* and *green* in order of their corresponding increasing cluster rank). Boutons outside clusters are marked in *grey*. For simplification and further quantification ellipsoids were fitted to each individual bouton cluster, denoted by ellipses shown in 2D view. The first neuron’s soma (ID 5) and its cluster centers are located close to the border of layer 1/2. The soma of the second neuron (ID 36) and its cluster centers are in layer 3. **b** Normalized depth from surface (*y-axis*) for each of the 50 neurons: 45 superficial pyramids (*grey dots*) and 5 layer 4 star pyramids (*grey pluses*). The neurons are sorted by their increasing depth from surface giving each neuron a unique ID between 1 and 50 (*x-axis*). The brain surface is at depth zero and the border of layer 3/4 is marked with a *dotted line* (border layer 3/4 = 1). **c** The depth from surface of each clusters ellipsoid center (*y-axis*) is individually plotted for each of the 50 sorted neurons (*x-axis*). The depth from surface is measured as the shortest distance between the ellipsoid center and the 3D brain surface. Each *colored dot* represents one cluster of a certain rank (see color-table). The *arrows* point to the shown example neurons 5 and 36. The *dotted grey* regression line highlights a significant correlation between the neurons ID and the distance from surface of its individual ellipsoid centers (*y* = 4.3*x + 1.2e + 002, Pearson correlation = 0.65, *p* value ≪ 0.05) (Neuron ID: 5, cat_0108_RH_neuron_04; 36, cat_0308_LH_neuron_01). *Scale bar* 1 mm

The cell body location (CBL) correlates with the number of boutons formed in each layer (see Fig. 3). Thus we investigated whether the individual clusters follow a similar pattern. We measured the shortest radial distance from the pial surface to the center of the ellipsoid fitted to each individual cluster (exemplified in Fig. 9a for the rank 3 clusters) and compared it to the neuron’s CBL (see Fig. 9b). This radial distance from pia to cluster centre was between 34 and 488 µm (mean 231 ± 92 µm), and indeed correlated with the CBL (Pearson correlation = 0.65, *p* value < < 0.05). Figure 9c illustrates the radial distance of the cluster with respect to the neuron’s CBL. The correlation was preserved when the radial distances were normalized to the thickness of the superficial layers (Pearson correlation = 0.63, *p* value < < 0.05).

### Formation of distal clusters by individual branches

**Fig. 10:**
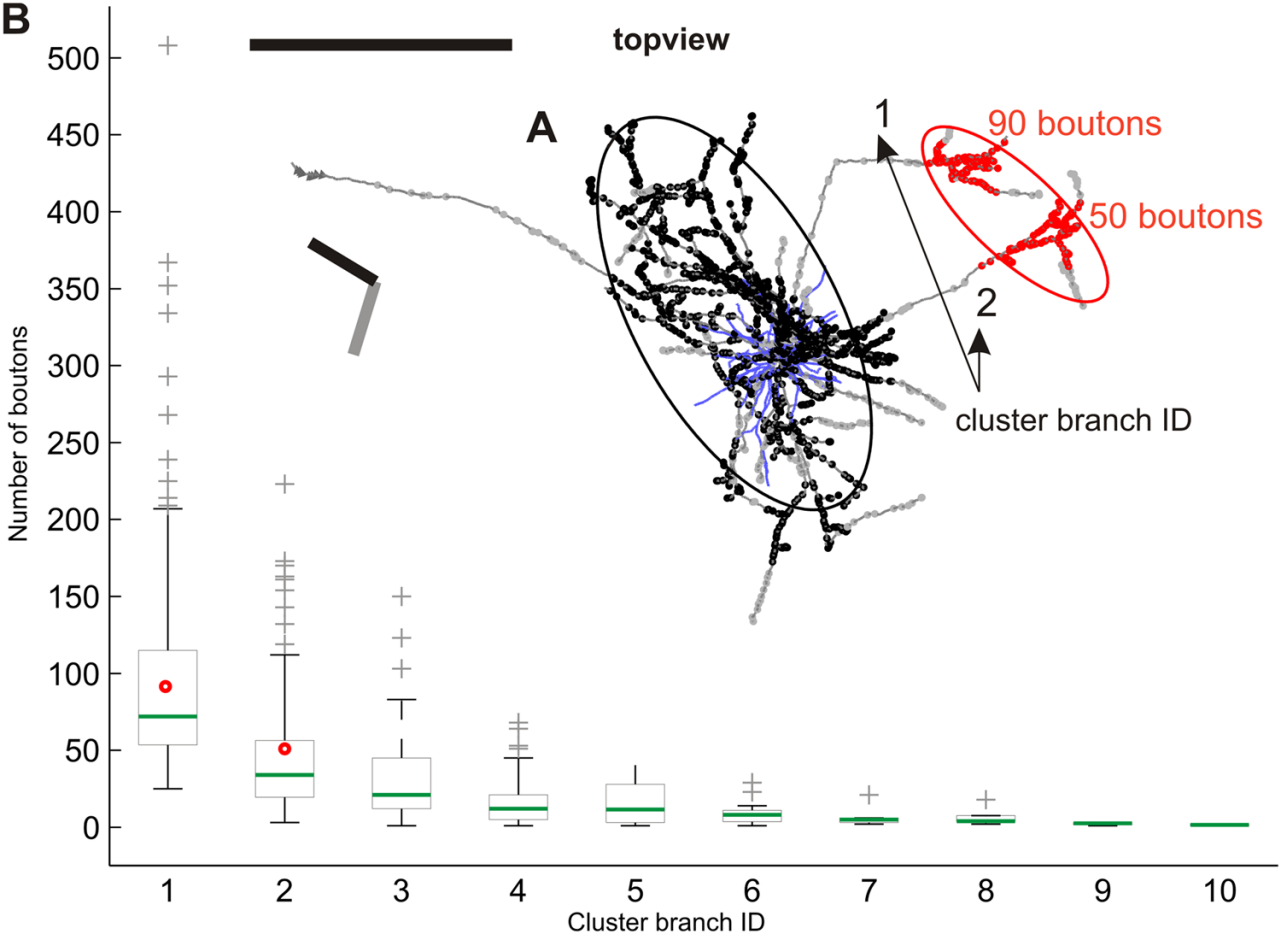
Number of boutons a certain cluster branch contributes to a distal cluster. **a** Example of a layer 2 and 3 pyramidal neuron (ID: 30). The neuron is shown in its top view together with the vertical meridian (*black bar*) and the neuron’s preferred orientation (*grey bar*). Boutons belonging to either the local or the single distal cluster are *color-coded* by *black* and *red dots*, respectively. Boutons outside clusters are marked in *grey*. For simplification and further quantification ellipsoids were fitted to each individual bouton cluster, denoted by ellipses here shown in 2D view (see “Methods”). The two axonal branches (1 and 2) that form the distal cluster are enumerated. This number denotes the *cluster branch ID* of each branch. The number of boutons each branch contributes is shown in *red* (90 and 50 boutons). In this example the branch contributing 90 boutons gets the cluster branch ID ‘1’, the other branch contributing 50 boutons gets the cluster branch ID ‘2’. **b** *Boxplot* for each of the cluster branch IDs and their comprised number of boutons, pooled across all distal clusters of all 50 neurons. *x-axis* Cluster branch IDs ascending sorted by their descending number of boutons they contributed to each individual distal cluster. The highest encountered cluster branch ID for one single distal cluster across all 50 neurons was ‘10’. *y-axis* Number of boutons one single cluster branch contributes to its cluster. *Boxplot* the *black box marks* the lower and upper quartile, the *green bar* the median, the *whiskers* extend in both directions till the ×1.5 interquartile range and outliers are marked with *grey pluses*. The *two red dots mark* the two cluster branches forming the red cluster of neuron 30 (**a**). Notice the unequal number of boutons different cluster branch ID’s contribute, which can be represented by an exponential distribution [*Y* = A*exp(B*X), with coefficients (with 95% confidence bounds): *A* = 164.6 (132.4, 196.9), *B* = −0.5731 (−0.7117, −0.4345)] (Neuron ID: 30, cat_0408_LH_neuron_01). *Scale bar* 1 mm

It was apparent from the 3D reconstructions that individual clusters could be formed by more than one collateral branch, as seen in the example in Fig. 10. Binzegger et al. (2007) reported an exponential decay when the number of boutons formed by different clusters is plotted against the rank of clusters weights. One mechanism is that the neuron forms its clusters according to some limited resource that is shared out exponentially by the neurons. We thus investigated how multiple collaterals that contributed to a single distal bouton clusters shared out their boutons. Did they simply provide equal numbers of boutons to the clusters or unequal amounts? The local clusters were excluded from this analysis since their axon of origin sits within the cluster itself. Individual distal clusters were formed by 1–10 contributing branches (see *x*-axis Fig. 10). The mean number of boutons any one branch contributed to a distal cluster was 52 ± 61, but the range was enormous (2–508). We thus examined how two or more branches placed their boutons to the same particular cluster. The branches were ranked according to the number of boutons they contributed to a distal cluster. The example neuron plotted in Fig. 10a shows that the first branch contributed 90 boutons and the second branch 50 boutons (see two red dots in graph) to the same cluster. When analyzed across all 50 neurons, an exponential decay was observed (see Fig. 10b), starting with a median of 70 boutons from the first branch, decaying further until the tenth branch, which made only 2 boutons (*Y* = A*exp(B*X), with coefficients (with 95% confidence bounds): *A* = 164.6 (132.4, 196.9), *B* = −0.5731 (−0.7117, −0.4345). The same decay was observed when taking the fraction of boutons each branch contributed to a cluster, instead of the absolute number of boutons. Thus, the more branches contribute to a single cluster, the lower the fraction of boutons from the first ranked branch. This was true when pooling all clusters with equal number of contributing branches and when pooling all clusters with equal ranks.

### The origin of distal clusters

**Fig. 11:**
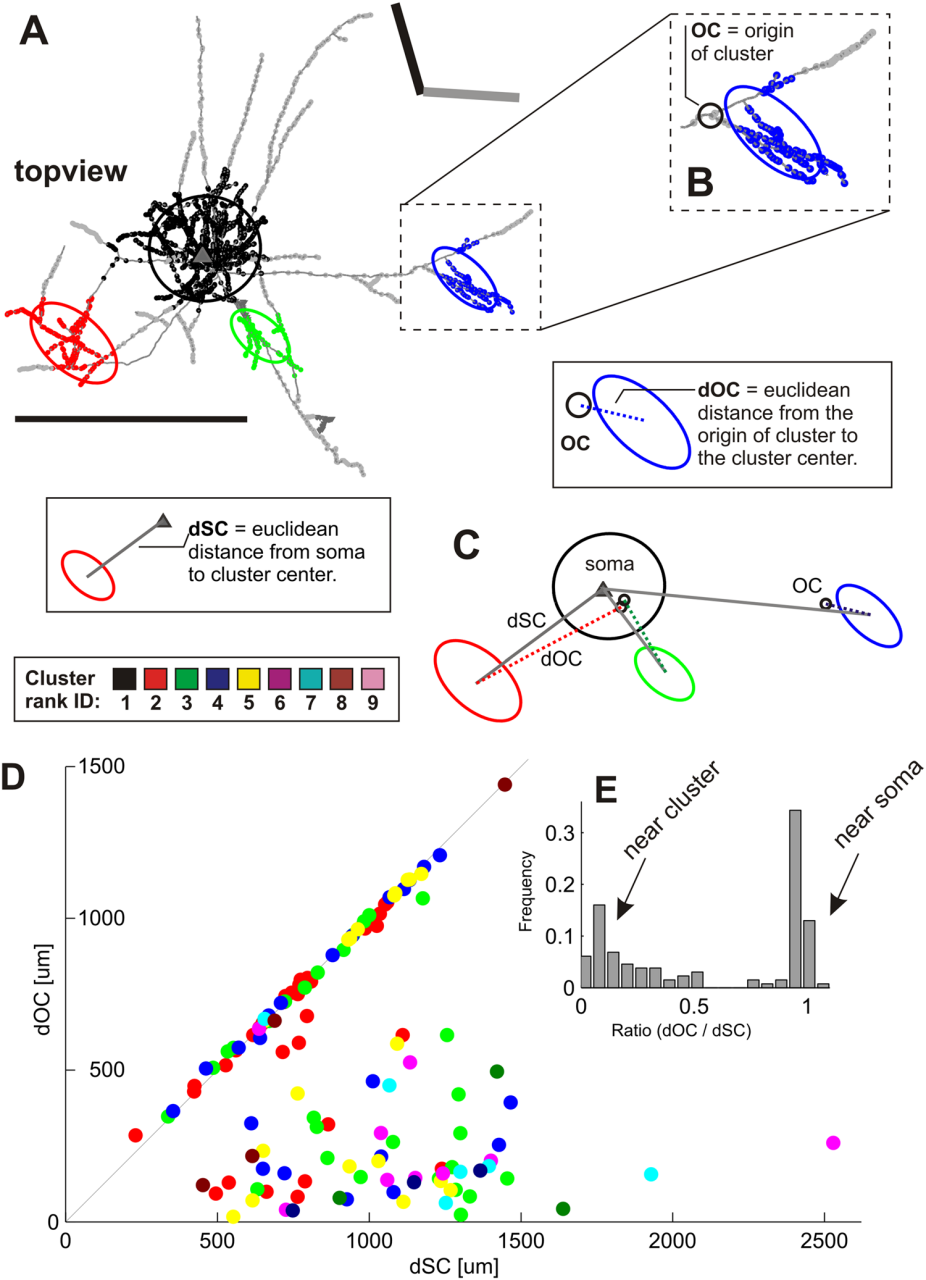
The origin of superficial layer distal clusters. **a** Example of a layer 2 and 3 pyramidal neuron (ID: 37). The neuron is shown in its top view together with the vertical meridian (*black bar*) and the neuron’s preferred orientation (*grey bar*). Boutons belonging to different clusters are color-coded by different colors (*black, red, green* and *blue* in order of their corresponding increasing cluster rank). Boutons outside clusters are marked in *grey*. For simplification and further quantification, ellipsoids were fitted to each individual bouton cluster and denoted as ellipses in the 2D view (see **c**). The *dashed rectangle marks* the region of the axon that has been magnified for *panel* **b**. **b** Higher magnification of the blue distal cluster with its fitted ellipse and the branches forming that cluster. The cluster is composed of a few branches that can be traced back to one unique location, called the ‘*origin of cluster*’ (=OC, orbited with a *black circle*). The OC is the closest site of a superficial layer distal cluster from which all its contributing branches originate (not to mistake for the origin of the axonal tree at the soma which is the origin of the whole axonal tree). **c** *Top view* of the individual ellipses of the same neuron color-coded by their individual cluster ranks. Each cluster’s OC is indicated with a *small black circle*. Notice that the *blue* cluster got an OC close to the cluster itself, whereas the *red* and *green* cluster got a OC close to the soma (clearly visible when tracing back for example, the axonal branches in **a** which contribute to the *red cluster*). The Euclidean distance from the OC to the cluster center is called dOC (*distance to origin of cluster*) and shown as *dotted lines*. The dOC of the *blue cluster* is exemplified in the inset. On the other hand, the *solid colored lines* represent the Euclidean distance from the soma to each cluster center (dSC *distance between the soma and the cluster*). The dSC of the red cluster is exemplified in the inset. Thus, each cluster has its unique OC, dOC and dSC. **d** Plot of the dSC (*x-axis*) versus the dOC (*y-axis*) from every individual superficial distal cluster across all 50 neurons. The *dots* are *color-coded* according to their individual cluster rank. Points falling along the bisector (*black line*) are clusters with similar lengths of their dSC and dOC, meaning that their OC is close to the soma (e.g., as for the *red* and *green* cluster of **c**). Points close to the *x-axis* got a small dOC, thus these clusters got an OC close to their cluster center (e.g., the *blue* cluster in **c**). The few points between the bisector and the *x-axis* represent clusters that got an OC between the soma and the cluster center. **e** Histogram of the ratio (dOC/dSC) of all points in **d** and their frequency. Most clusters got their OC either close to the soma (ratio near 1) or close to the cluster center (ratio near 0) (Neuron ID: 37, cat_0707_RH_neuron_01). *Scale bar* 1 mm

The above analysis has shown that a single cluster can be innervated by more than one collateral branch. This is clearly visible when looking at all the top-views of individual bouton clusters and their contributing branches (see for example Figs. 1f, 6a, b, blue clusters). As each distal cluster had a different pattern of contributing branches, we were interested in identifying the location of the single branch from which all resulting daughter branches made up one distal cluster. In other words, we wanted to find the cluster’s closest parent node and identify where that parent node was located on the axonal tree: was it near the cluster itself or near the parent soma? To answer this question, we identified the parent node as the Origin of Collaterals (OC). Figure 11a, b illustrates the concept of the OC. Having identified the OC, we then measured the distance from the OC to the cluster center (Fig. 11b inset). This distance was termed as the distance of the OC to the cluster, i.e., dOC (Fig. 11b inset). In a third step we measured the distance from the parent soma to the OC, abbreviated as dSC (Fig. 11c inset). With these 3 parameters (OC, dOC and dSC; see Fig. 11c), we were able to determine where the parent node was located on the axonal tree in respect to the soma and cluster center.

We extracted the dOC for all 217 distal clusters across the 50 neurons and observed large variations (range 43–1440 µm, mean 546 ± 349 µm). When plotting the dOC versus the dSC (see Fig. 11d), many data-points lay on the diagonal, indicating that many distal clusters had their origin (OC) very close to the soma. This in turn means that many distal clusters are formed by collateral branches that originate near the soma and then travel individually through the neuropil before converging to form a single distal cluster. The data in Fig. 11d revealed the existence of a second population of clusters that had their origin (OC) near the cluster center (i.e., along the *x*-axis and hence small dOC), which means that these clusters are formed by collaterals that branched close to the cluster itself.

These two strategies of collateral branching (OC near cluster center or OC near soma) are revealed in Fig. 11e, where we calculated the ratio between a clusters’ dOC and a cluster’s Euclidean distance to the soma (dSC). This produced a bimodal distribution in which 53% of all distal clusters had a ratio higher than 0.9 and 33% of all distal clusters had a ratio less than 0.3. The high ratios were indicative of branching near the soma, the low ratios that the OC was close to the cluster itself. Only very rarely was the origin of a cluster in the middle of one of the ‘spokes’ that connected the main axon to the distal clusters.

### Tortuosity of branches forming distal clusters

While following single axonal branches, which traveled millimeters through the neuropil to form the distal clusters, we observed that individual branches often did not reach their terminal cluster by the most direct route. Instead branches made loops to their target region, as if the initial growth was not very precise and then gradually had to be redirected onto their ‘right’ location (see for example Figs. 1f, 6b, blue cluster, Fig. 10a red cluster). To quantify the tortuosity of axonal branches forming each cluster, we divided its Euclidean horizontal distance by the 3D axonal length from the soma to the cluster center. A fraction close to 1 signifies that both lengths were equal, whereas a small fraction signifies that the distance along the axon was much longer compared to the horizontal distance. The average tortuosity measured 0.76 ± 0.09 (0.55–0.98) indicating that single axonal branches forming distal clusters are on average 1.32 times longer compared to the Euclidean distance.

### Summary of principal findings

**Fig. 12:**
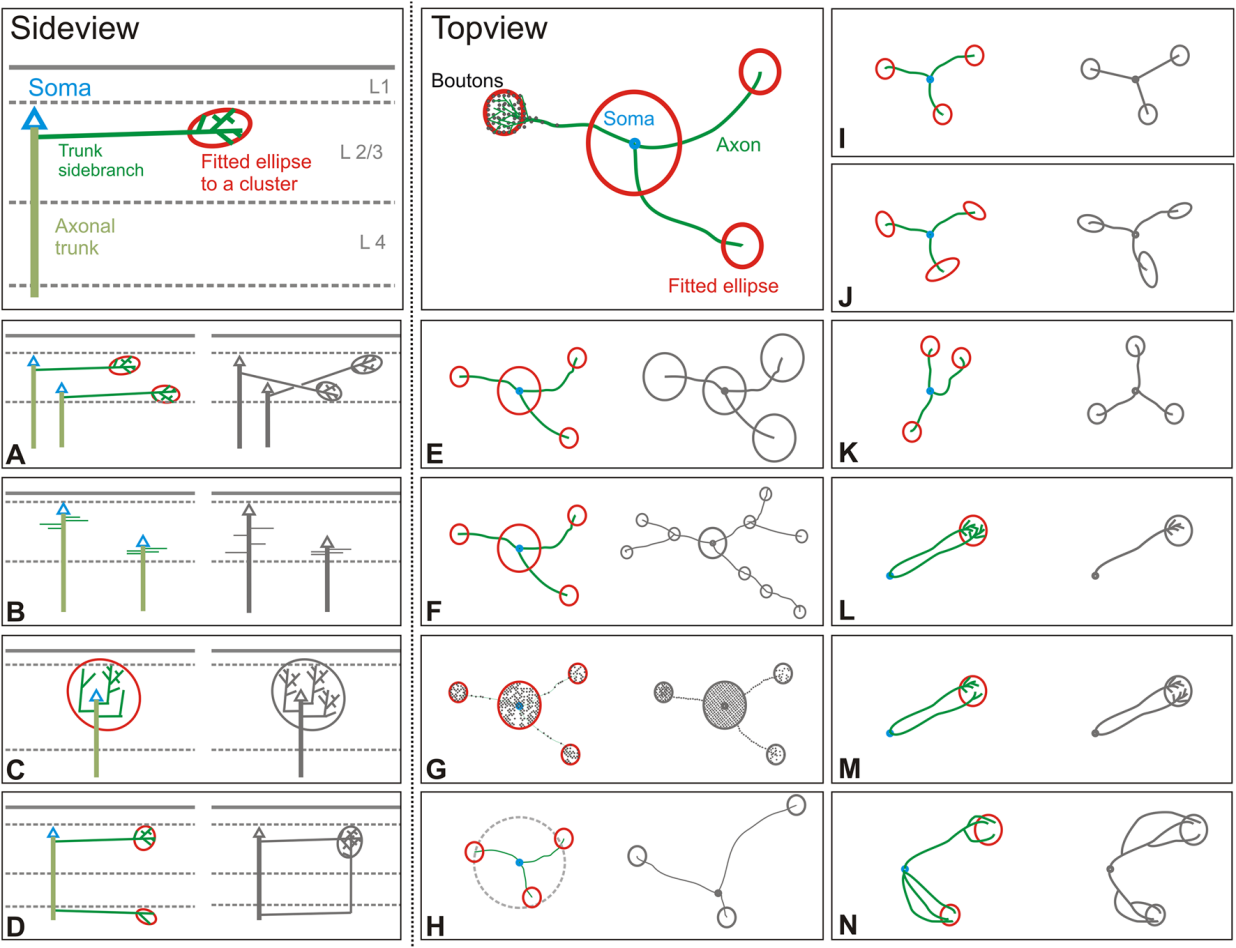
A biological blueprint of superficial layer pyramidal neurons. The rules illustrated in **a**–**n** are based on our morphological and topological analysis from 50 intracellularly filled and subsequently 3D-reconstructed superficial layer pyramidal neurons. The colored sketch on the *left* of each subplot (**a**–**n**) depicts what we commonly observed in each individual analysis whereas the sketch in grey on the right displays the unusual or rare cases. **a–d** denote side views of schematic neurons and their clusters, whereas **e**–**n** show top views. **a** The innervation within the superficial layers depends strongly on the neurons individual cell body location (CBL). The CBL determines as well the cluster center’s depth from pial surface. Additionally, neurons maintain a narrow sub-layer specificity of their axons, concurrently creating fine-grained distribution of boutons and clusters across the superficial sub-layers. **b** Each axon comprises a main trunk that projects radially to white matter. Branches from the trunk are called ‘trunk sidebranches’ (TSB). On average the first TSB is formed after 74 µm and after 142 µm half of all TSBs are established, independent of the neuron’s CBL. **c** The first TSB contributes the highest fraction of boutons to the local cluster. **d** Clusters in superficial layers are made by TSB’s bifurcating within the superficial layers. **e** Local and distal clusters are eloquently different in terms of the number of boutons they contain (local 1079, distal 166) and the mean geometric diameter (local 511 µm, distal 256 µm). The local cluster comprises 70% of all clustered boutons whereas one distal cluster comprises 10%. **F** One neuron forms on average three distal clusters and always one local cluster. Radial branches contribute to one distal cluster and form rarely a second vestigial cluster along the same radial direction. **g** The bouton density is equal for all clusters: the local cluster has 2 boutons and the distal clusters 2.5 boutons per 50 × 50 × 50 µm volume. The interbouton interval (IBI) is for clustered regions 65% lower than for unclustered regions. **h** The distal clusters are located at a mean radial distance of 942 µm form the soma with a standard deviation of 332 µm. This creates an annulus in which distal clusters can be encountered. **i** Single axonal branches do not grow straight to their target region. The tortuosity of 0.76 indicates that the path along an axon towards a distal cluster is 30% longer than the straight horizontal distance to the identical distal cluster. **j** Individual distal clusters are not soma eccentrically elongated. **k** Individual clusters are circularly randomly placed. **l** 1–10 single branches meet to form one individual distal cluster. **m** The fraction of boutons each branch contributes to a distal cluster decays exponentially with the number of contributing branches. Two branches rarely contribute the same amount of boutons. **n** The ‘origin of cluster’ (OC) is the next closest common node to a cluster to which all axonal segments forming that specific distal cluster can be traced back. The OC was either close to the soma or close to the cluster center and rarely between

Our quantitative analyses of the axons and their bouton clusters allowed us to identify many characteristics that describe the axon arborizations of the superficial layer pyramidal neurons in cat V1 (summarized in Fig. 12). The schematics in color (left columns) indicate the typical case, while the schematics in grey indicate alternative possibilities that were rarely or never observed.

## Discussion

In higher mammals the functional cortical ‘columns’ are interlinked by lateral excitatory connections that form a patchy network known as the cortical ‘daisy’ because of its appearance in tangential sections (Douglas and Martin 2004). However, even after decades of investigation, a high-resolution qualitative and quantitative description of the single neurons forming the ubiquitous, if enigmatic, cortical daisy remains elusive. The analyses of 50 intracellularly filled pyramidal neurons in the superficial layers of the cat’s primary visual cortex indicate that single neurons establish highly individualized single lateral clusters: no two neurons appear the same. Nonetheless there are a surprising number of common features that are expressed in the structure of the axonal arborizations. Identifying these common features allowed us to outline a comprehensive description of the arborizations (illustrated graphically in Fig. 12) of the superficial layer pyramidal neurons—the elemental building blocks of the cortical daisy.

We confirmed previous studies that showed the existence of distinct regions of the axon (Ojima et al. 1991; Yabuta and Callaway 1998; Kisvarday et al. 1986; McGuire et al. 1991; Martin and Whitteridge 1984; Binzegger et al. 2007). The axons form one local cluster and several distal clusters of boutons. Linear segments of axon with relatively few boutons link the distal clusters to the main axon. Our analyses indicate that there is one set of relatively constant parameters (e.g., sublamination, stereotype main trunk and its branches, equal bouton cluster density, similar horizontal and gradual vertical distance of clusters) and another set of variable parameters (e.g., size of clusters, number of distal clusters and number of contributing branches, non-soma-eccentric cluster elongation and circular irregularity, tortuosity). We have shown that at least some of these variable parameters nonetheless follow clear patterns, perhaps the most striking of which is the exponential allocation of boutons across the various clusters.

Besides the current description of these *structural* parameters, we performed recently a fine-grained *functional* analysis where we looked at the relation between the orientation map and axonal arborizations for a subset of 33 of the 50 neurons studied here for which we were able to obtain intrinsic optical imaging recordings of the orientation map (Martin et al. 2014). We calculated a similarity index (SI) that expressed the similarity of the orientation domain of a given bouton cluster to that of its parent dendritic tree. We observed a surprisingly high variation in the SIs across all distal clusters and even found clusters in orientation domains that were orthogonal to the parent cell’s preferred orientation. We searched here for correlations of a cluster’s SI value with the various parameters investigated here (i.e., cluster size, cluster diameter, TSBs, IBI, horizontal and vertical distance), but found none. In the next few sections we discuss the implications these *structural* and *functional* observations have on the cortical daisy.

As previous studies have shown, the majority of bouton clusters were located in the superficial layers (Rockland and Lund 1982; Binzegger et al. 2004; Levitt et al. 1994) with a smaller, but consistent innervation of layer 5 (Martin and Whitteridge 1984; Gilbert and Wiesel 1979; Gilbert 1983; Stepanyants et al. 2009; Binzegger et al. 2004, 2007; Karube and Kisvarday 2011; Buzas et al. 2006). In the current study, we discovered a distinct sublaminar organization of the clusters that had not been detected in previous studies, probably because so few such neurons have been reconstructed in 3D until now. We found that the laminar depth of the clusters was positively correlated with the depth of the cell soma, which suggests the cell and the cluster boutons might use the same depth positional signal in development. This sub-lamination is not explained by the positions along the main axon trunk where the collaterals that form the clusters branch, since we showed here that the branches are emitted at highly stereotyped distances from the soma, regardless of the soma’s depth. This is supported by development studies in the cat (Galuske and Singer 1996) who observed that the developmental time at which initial bifurcation from the main trunk occurred did not depend on the laminar position and maturity of the neurons, and by Ojima and colleagues who described such a main trunk in cat auditory cortex (Ojima et al. 1991). It is well-known that different receptive field types reside in different laminae of cat V1 (Hubel and Wiesel 1962; Gilbert and Wiesel 1979), so searching more carefully for a sublaminar organization within the cortical daisy with electrophysiological or optical methods may yet be revealing.

Another remarkable observation was that the 1–10 branches forming collectively a single cluster kept the bouton density in a relatively narrow range between 16 and 20 K boutons/mm^3^, regardless of the individual number of boutons contributed by each single branch or the overall volume of the cluster. In addition, we also confirmed that not only were the number of boutons in a cluster exponentially distributed relative to its rank (Binzegger et al. 2007), but that individual branches feeding into a single cluster also distributed their boutons exponentially in a similar fashion. Thus, by taking these two exponential rules together, we can predict how overall the boutons will be distributed across the individual clusters (if the total number of boutons and number of clusters is known), and we can predict the number of boutons each successive branch will contribute to one specific cluster (if the total number of boutons within a single cluster and the number of branches forming the cluster is known). Such clear exponential relationships are not immediately obvious at the single neuron level and only emerge upon quantitative analyses.

We confirmed the finding of Binzegger et al. (2007), that on average 70% of the total number of boutons lie within the local cluster. This is a significant finding in view of the claim by Stepanyants et al. (2008) that 92% of boutons formed by superficial layer pyramidal cells in cat visual cortex lie outside a 200 µm diameter iso-orientation column, centered on the soma. Even for an ocular dominance column of 800 micron diameter, they estimated that 76% of the boutons lie outside the column. The reason for this very large discrepancy is due to their assumption that a cylinder is the appropriate shape to describe a functional column. It is clear from functional mapping that orientation and ocular dominance columns are not cylinders (see review da Costa and Martin 2010), nor is the local cluster. Thus the procrustean approach of Stepanyants et al. (Stepanyants et al. 2008) is not well matched to either the architecture of functional maps, or to the actual structure of the axonal arbors. In our study of the relation of single axonal arbors to the orientation map, we demonstrated that the elliptical local bouton cluster occupies orientation domains that are very similar to those occupied by the dendritic tree (Martin et al. 2014). Hence, by taking into account the actual shape of the local cluster and the iso-orientation domains, we found there is a logical match of structure and function.

Cortical axons are best described as tree structures (Anderson et al. 2002; Binzegger et al. 2005, 2007) and this constrains how they lay down their boutons. For example, we showed here that the linear segments that form the distal clusters do not branch until they reached their final location to form a cluster. In a previous study (Binzegger et al. 2005), we determined the complexity of axonal arborizations in cat V1 and discovered that the major contribution to the complexity of superficial layer pyramidal neurons came from the branching within the clusters. This degree of branching complexity is also relevant to the questions of wiring efficiency and associated conduction times through the axon.

In their study of wiring ‘cost’ of either physical length of ‘wire’ or of conduction delays, Budd et al. (Budd et al. 2010) calculated that the pyramidal cell axons were about 14% longer than the theoretically shortest axon that would link the soma to all boutons. This is a modest increase in length, but it did not take into account the fact that multiple branches arborize near the soma and travel all the way from the soma to arborize in the same cluster. We also found that the axon connecting the soma to the distal cluster was 30% longer on average than the Euclidean distance between them. Thus, to link all boutons by the route that would minimize conduction delays would lead to very significantly increase the grey matter volume (Budd et al. 2010). It is clear from our analysis of superficial layer pyramidal cell axons (Binzegger et al. 2005) that the distal clusters contribute a disproportionate amount of the total axon length of nearly 50 mm, due to the fan-out of bouton-laden branches. Our observation that clusters were formed between 1 and 10 lateral collaterals indicates that neither wire minimization nor the temporal dispersion caused by different routes of transmission leading to the same target are especially hard constraints on the optimization of cortical wiring.

That a neocortical pyramidal cell has a general intrinsic developmental program that leads to identifiable principles of axonal organisation across all investigated neurons as we illustrate in Fig. 12, is unsurprising. It does not mean; however, that the cell generates its phenotype independently of any other cell. Clearly the very existence of the cortical daisy points to the existence of cooperative mechanisms whereby the cells contributing to the daisy ‘agree’ on the size of the collective bouton cluster, the distance between clusters, and the bouton density within a cluster. Thus, while individual neurons do not contribute boutons to all the clusters of a daisy, individual neurons participate in a collective process that generates the regular hexagonal arrangement seen in top view of the daisy (Muir et al. 2011). This is supported by the simulations of Muir and Douglas (Muir and Douglas 2011), who concluded that the clustered projections of the daisy cannot develop solely using information intrinsic to single neurons. The placement of clusters is indeed non-random (Martin et al. 2014) and thus the result of a deliberate wiring strategy. The extensive list of constant and variable parameters we discovered are the reasons that single neurons form each of their lateral clusters in a highly individualized manner, yet retain their strong family resemblances.

The observed average distance from soma to the distal clusters of about 1 mm has a double relevance functionally. *First*, per linear mm of cat cortex, it is known that the magnification of the central visual field is 0.5°–1.0° of visual field (Rosa et al. 1995; Tusa et al. 1978) and the receptive fields (RF) of most neurons in layers 2 and 3 are only a few degrees on a side (Gilbert 1977). This means that the distal clusters described here form monosynaptic connections with neurons whose receptive fields overlap with the neurons in the domain of the local cluster. The distal clusters do not form the surround of the receptive field, as is often supposed (see review by Bouscein et al. (Boucsein et al. 2011). Instead the evidence is that they change the gain of the response of the classical receptive field center in a context-dependent fashion (Girardin and Martin 2009; Martin et al. 2014). *Second*, Hubener et al. (Hubener et al. 1997) observed in cat V1 a center-to-center spacing of one mm for three different stimulus attributes (orientation, ocular dominance, and spatial frequency). Our data indicate that a neuron is potentially able to reach any other neuron within the 1 mm radius by means of monosynaptic connections. Generally, the individual distal clusters serve simultaneously two key mechanisms: They permit crosstalk between neurons having overlapping receptive fields and they allow traffic between domains of different stimulus specificity. Thus, instead of treating the daisy as single homogenous network, the real clue to interpreting its function may lie in its structural heterogeneity, which allows different functional domains to influence each other. This seems to be an appropriate architecture for a computational network for context-dependent processing of natural scenes (Martin et al. 2014).

## Methods

### Surgery

The neurons were collected from 15 anesthetized adult cats of both sexes (19 hemispheres) that had been prepared for in vivo intracellular recording. All experiments were carried out with authorization and under license granted by the Kantonalem Veterinaeramt of Zurich. The cats were prepared for surgery after the administration of subcutaneous premedication of xylazine (Rompun, Beyelar, 0.5 mg kg^−1^ and Narketan 10, Vetoquinol AG, CH, 10 mg kg^−1^). Initial surgery was performed under additional gas anesthesia using 1–2% halothane (Arovet AG, CH) in gen/nitrous oxide (50%/50%). After induction of general anesthesia the femoral vein was cannulated and alphaxalone/alphadalone (Saffan, Glaxo) was delivered to establish complete anesthesia during the remainder of the experiment. The femoral artery was cannulated to measure blood pressure. After a tracheotomy the cat was moved to a stereotaxic apparatus, where it was respirated artificially with a mixture of oxygen/nitrous oxide (30%/70%). Halothane (0.5–1.5%) supplemented the i.v. anesthesia when needed. The respiratory pump volume was adjusted to constant 4.5% end-tidal CO_2_. Lidocaine gel (4%) was applied to all pressure points. Electroencephalogram (EEG), ECG, heart rate, blood pressure, end-tidal CO_2,_ and rectal temperature were monitored continuously during the entire experiment. A thermistor-controlled heating blanket maintained the cat’s rectal temperature at 37 °C. Topical antibiotics (Voltamicin, Novartis) and Atropine 1% (Novartis) (to paralyze in accommodation) were applied to the eyes before they were covered with gas permeable contact lenses. To retract the nictitating membrane phenylephrine 5% (Blache) was used. A craniotomy was performed over area 17 from Horsley–Clark coordinates AP-1 to -9 and LM from the midline to 5 mm lateral. A plastic chamber was mounted over the craniotomy and fixed to the bone with dental cement. After the craniotomy the cats received an intravenous injection of the muscle relaxant gallamine triethiodide (40 mg induction dose) (Sigma Aldrich, CH) followed by a continuous infusion of gallamine triethiodide (13 mg kg^−1^ h^−1^) and (+)-tubocurarine chloride hydrate (1 mg kg^−1^ h^−1^) (Sigma). The eyes were refracted and lenses were added to focus the eyes on a tangent screen.

### Recording and HRP injection

Glass micropipettes were filled with a 4% solution of Horseradish Peroxidase (HRP, Roche) in 0.05 M Tris and 0.2 M KCl at pH 7.9 and then beveled to impedances between 40 and 88 MΩ (mean 72 ± 12 MΩ). A small durotomy was made for each penetration. The location of the penetration was noted on a drawing of the pattern of blood vessels on the surface of the cortex. Micropipettes were lowered to brain surface then the chamber was filled with agar (Sigma) in Ringer solution and sealed with paraffin wax.

After hand-plotting of the extracellular receptive fields (RFs), an attempt was made to impale the neuron by advancing the micropipette in 2 µm intervals while passing current pulses of 2 nA. A drop of the DC potential (from −40 to −70 mV) and a large increase of the amplitude of the action potential indicated that the micropipette successfully penetrated the membrane of a neuron’s cell body. After entering a neuron’s soma its RF was checked to be sure that the extracellularly identified RF belonged to the actual neuron (it invariably did). Then HRP was injected by iontophoresis of positive pulses of 4 nA in a 200 ms ON/50 ms OFF duty cycle for a duration that ranged from 10 to 180 s (mean 60 ± 33 s) (for details see (Martin and Whitteridge 1984). The relationship between the physiology and the anatomy of this set of single neurons was discussed previously (Martin et al. 2014) and thus physiological properties were not analyzed in this study.

### Fixation and histology

At the end of the experiment the cat was given an overdose of anaesthetic until the EEG became flat. Then the cat was perfused transcardially with normal 0.9% NaCl solution, followed by a room-temperature solution of 4% paraformaldehyde, 0.3% gluteraldehyde and 15% saturated solution of picric acid in 0.1 M PB pH 7.4. After perfusion the block of brain containing the relevant piece of area 17 was removed and washed in 0.1 M PB for at least 2 h to remove remaining fixative. To cut the tissue block containing area 17 horizontally it had to be further trimmed. The whole block was embedded in agar, sectioned at 80 μm (MICROM HM 650 V) in the horizontal plane, collected and washed several times in 0.1 M PB. The HRP activity was identified using 3-diaminobenzidine tetrahydrochloride (DAB) with nickel intensification. After assessment by light microscopy (LM) the sections were further processed for electron microscopic analysis. These sections were treated with 1% osmium tetroxide in 0.1 M PB, dehydrated through alcohols (1% uranyl acetate in the 70% alcohol) and propylene oxide, and flat mounted in Durcupan (Fluka) on glass slides.

### Neuron reconstructions

Neurons were reconstructed in 3D using a Microscope (100x, Olympus BX-51) combined with a motorized stage (MicroBrightField Inc. USA) and the aid of the Neurolucida software (Version 8.0, MicroBrightField Inc. USA). Before starting the reconstruction of the individual neurons a 3D scaffold was generated. This scaffold contained the individually aligned tissue sections using reference penetrations and various fiducial marks (e.g., blood vessels). Additionally, section outlines were drawn to highlight the brain surface. This scaffold enabled a very precise alignment of the individual sections, which assisted the subsequent tracing of the various neurons and their fine axonal processes over several tissue sections. The time consuming reconstruction of one neuron took approximately 100 h. While reconstructing the axon each encountered bouton was tagged with a marker.

Each neuron contained two regions of bifurcation zones, one in the superficial layers and one in the deep layers. The one axonal segment, which connected the superficial and the deep part of the axonal tree, was tagged with an extra marker called ‘OT5’. This marker was necessary to differentiate between the superficial and the deep part of the axonal tree. As a final step the dendrite was reconstructed in 3D.

Further all layer boundaries were digitized and saved in the scaffold. The borders of cortical layers were determined in tangential sections on the basis of light microscopic characteristics visible in the osmium-treated tissue, such as relative neuron and fiber densities, neural soma size, HRP-filled dendrites, the presence of large pyramidal cells at the border region of layers 3 and 4 and giant pyramidal cells of Meynert in layer 5b.

### Preprocessing

The reconstruction software Neurolucida has limited tools for data analyses. Therefore, a framework was created in house called ‘Nereda’. With this framework the substantial number of neurons could be analyzed down to the smallest details in reasonable computing time. Before doing any analysis, the scaffold (i.e. one xml data-file containing all relevant data for one single neuron) was used for two crucial preprocessing steps: (1) creating volumes out of the layer boundaries and (2) applying a mean-shift bouton clustering to each individual neuron.

1. Layer boundaries: The layer boundaries resembled contour lines of a hill in a topographic map. To quantify any particular layer affiliation in 3D these lines had to be converted into volumes. The 2D lines of the different layers were first converted into surfaces by the aid of a triangulation tool developed by Dr. Dylan Muir. This triangulation implied a virtual ball of a certain size rolling over the contour lines thus creating a triangulated surface. This triangulated surface created a subjacent volume, which was further discretized by applying a voxelization (voxelsize 20 µm). Now datapoints could be affiliated in 3D to a certain voxel which themselves belonged to a certain volume under a specific layer boundary. For example, each individual bouton of one axonal tree could be attributed to a different layer by first assigning the bouton to an individual voxel in 3D.
2. Bouton clustering: The three-dimensional arborization pattern of an axon is typically heterogenous, composed of spatially separated regions of axonal arborizations and bouton formation. These “patches” have a high bouton density relative to the surrounding zone, and the density distribution, therefore, resembles a landscape, where hills indicate regions of high bouton density, and the different regions are delineated by low density plains or valleys. Binzegger et al. (Binzegger et al. 2007) investigated these regions of high bouton density or clusters and developed a mean-shift cluster-algorithm, which was applied to each neuron (for details see Binzegger et al. 2007). Briefly, a smooth density landscape was obtained by convolving the bouton locations with a spherical Gaussian kernel of width *h*. Each local maximum of the density landscape is a peak of a hill and the set of boutons forming the hill defines the cluster. To determine which boutons belong to the hill, boutons were moved along the local gradient until a local maximum was reached and they could easily be identified.

The well-established “mean-shift” algorithm is an iterative procedure that performs these steps without the need to calculate explicitly the density landscape and the gradient. The mean-shift algorithm formed the heart of the clustering procedure, which involved the following three major steps: (a) eliminating the linear structures, (b) choosing the appropriate width *h* for the convolution kernel and (c) post-processing to exclude clusters.

1. Elimination of linear structures: Not all the boutons of an axon were contained in clusters. Every axon contained long-range isolated axonal branches, which in many cases extended over several millimeters and finally ended up forming a cluster. These linear branches were identified and reduced before the mean-shift algorithm was applied. To do so, the bouton cloud was partitioned into small local regions where each region was classified as being part of a bigger linear set of points L if (1) its boutons were formed by less than five branches, (2) the 3D ellipsoid fitted to the individual bouton cluster was highly elongated (0.08), (3) the region of the fitted ellipsoid was very small (<0.01 um^3^). Partitioning was done using the mean-shift algorithm with a spherical Gaussian kernel with width PHI. The set L will then depend on PHI where a large PHI will produce a small set L, and a small PHI will produce a large set L containing most of the boutons in the cloud. We chose PHI so that the fractal dimension of L was close to 1 (between 0.9 and 1.1). Boutons that felt into the cloud L were classified as linear and not further used for the clustering.
2. Choosing the appropriate width *h* for the convolution kernel: After the elimination of L, the remaining boutons were partitioned into clusters using the mean-shift algorithm with kernel width *h*. The choice of *h* controlled the smoothness of the density landscape. It can be thought as the equivalent of the bin width in histograms. A large *h* will result in a very smooth landscape where only the gross features are represented, and the smaller *h* is chosen, the more local maxima appear. The value of the parameter (*h*) was iteratively chosen that only the coarser grain clusters associated with the columnar systems of visual cortex (200–400 µm).
3. Post-processing to exclude clusters: Clusters obtained from the mean-shift algorithm with predominately inhomogeneous bouton arrangements were excluded from analyses. In particular, clusters containing only (1) a couple of branches (<5), (2) no boutons within the ALPHA-ellipsoid (ALPHA < 0.1) or (3) a small number of boutons (<50 boutons). Those clusters that were omitted in this post-processing step were termed as omitted clusters, all other clusters were named as regular clusters.

By applying the mean-shift algorithm to each individual neuron their boutons and axonal segments could explicitly be affiliated to one of the three compartments: (a) linear regions, (b) regular clusters or (c) omitted clusters (for further details see (Binzegger et al. 2007). For each neuron those compartments and their boutons were cached and could be retrieved in case of need.

Summarizing, for each neuron one scaffold was created containing the following data: the brain surface, cell body, axon, dendrite, boutons, the marker OT5, the layer volumes, and the bouton clusters. This scaffold was the basis for all further analyses. Figure 1 displays one typical superficial pyramidal neuron.

### Terminology

#### Bouton clusters

Basic cluster measurements were based on 3D ellipsoids that were fitted to regular bouton clusters (for simplicity we termed a 3D ellipsoid simply as *ellipsoid*). The ‘cluster center’ is the center of the ellipsoid. The ‘cluster diameter’ is defined as the geometric mean of the three diameters of the ellipsoid. The Euclidian distance between the cluster center and the soma is called the ‘horizontal distance’ of a cluster. The shortest distance from the brain surface to the cluster center is termed as ‘vertical distance’ of a cluster. The ‘cluster size’ is defined as the number of boutons it contains. The ‘cluster bouton density’ is measured by the ratio of the number of boutons in the ellipsoid and the volume of the ellipsoid. The ‘cluster weight’ is the ratio of the boutons in the cluster and the summed bouton number in all clusters. The association of the boutons to a cluster is indicated by different colors, where the color codes for the relative number of boutons each cluster contains (i.e., black indicates the cluster with the highest number of boutons, followed by red, green, and blue, etc.). We also refer to the cluster with the highest number of boutons as the rank 1 cluster, the cluster with the second highest bouton number the rank 2 cluster, and so on. The linear regions and the omitted boutons resulting from the preprocessing step were pooled together and termed as unclustered region and associated with the color grey. The regular clusters were further subdivided into distal clusters and one local cluster. The local cluster was the most proximal one to the soma and the remaining regular clusters were classified as distal clusters. Thus three new compartments were defined: The unclustered or linear region, the local cluster and the distal clusters to which we frequently refer in our analyses.

#### The vertical axis

A line through the axonal origin at the soma and the marker OT5 formed an axis termed as the ‘vertical axis’. It can be thought as the equivalent of the radial orientation of the microcolumn in which the neuron is located. The distance of the neuron’s cell body from the brain surface (or ‘cell body location’ = CBL) is measured as the distance of the vertical axis from the soma till the brain surface. In so far as we are concerned with the superficial clusters in all further analyses the axonal processes below the OT5 were excluded, except specifically stated.

#### The projection plane

Because the imaged region of area 17 was on the top of the gyrus representing the area centralis, we had to take into account the gyral curvature (Tusa et al. 1978). A ‘projection plane’ was created for each individual neuron. This projection plane was orthogonal to the vertical axis centered on the soma. The projection of an individual neuron onto their projection plane can be thought as an azimuthal projection, hence making analyses such as a 2D Sholl comparison between neurons meaningful. For some analyses we fitted a 2D ellipse to the bouton cluster for a single projection plane (for simplicity we termed a 2D ellipse simply as ‘ellipse’, so not to be confused with an ellipsoid).

#### The trunk

Each axon comprised a series of concatenated segments originating at the soma forming the shortest connection to the layer 5. This successional formation of segments is termed as the ‘trunk’ and resembles the vertical axis. The branches from the trunk are called ‘trunk side branches’ (TSB). The sketch drawn in Fig. 5 allows a better understanding of the trunk.

#### Origin of a cluster

The axonal tree as a whole has one origin located at the soma. Thus, the whole axonal tree can be traced back to that origin. Here, we were also interested in the origin of individual distal clusters. The ‘origin of cluster’ (OC) is the next closest common node to a cluster to which all axonal segments forming that specific distal cluster can be traced back. The OC can be located anywhere in the axonal tree between the soma and that specific cluster (see inset B of Fig. 11). Each OC has a particular Euclidean distance to its cluster center, called here the ‘distance between the origin of cluster and the cluster center’ (dOC). Figure 11 explains the concept of the OC and the dOC.

### Statistics

Extracted parameters are quoted by their means and standard deviations. The range of individual values is stated by its minima and maxima. To evaluate a linear relationship between two parameters we used the Pearson’s correlation with a significance threshold of 0.05. If a statistically significant linear relationship existed between two parameters the correlation coefficients *R* square (‘*r*’) is mentioned together with the critical *p* value. For each analysis we pooled the layers 2 and 3 pyramids and the layer 4 star pyramids in one group. If a significant difference was encountered between the star-pyramids and layers 2 and 3 it will be mentioned in that specific analysis. All parameters resulting from each analysis were always tested against all characteristics of individual bouton clusters (e.g., cluster rank, size, weight, and the horizontal distance) but only mentioned if a relationship was significant.
